# The advances in nanomedicine for bone and cartilage repair

**DOI:** 10.1186/s12951-022-01342-8

**Published:** 2022-03-18

**Authors:** Kai Qiao, Lu Xu, Junnan Tang, Qiguang Wang, Khoon S. Lim, Gary Hooper, Tim B. F. Woodfield, Guozhen Liu, Kang Tian, Weiguo Zhang, Xiaolin Cui

**Affiliations:** 1 Department of Bone & Joint, the First Affiliated Hospital of Dalian Medical University, Dalian, 116000 Liaoning China; 2 Department of Cardiology, the First Affiliated Hospital of Zhengzhou University, Zhengzhou, 450052 Henan China; 3 National Engineering Research Center for Biomaterials, Sichuan University, Chengdu, 61004 Sichuan China; 4 Christchurch Regenerative Medicine and Tissue Engineering (CReaTE) Group, Department of Orthopaedic Surgery & Musculoskeletal Medicine, University of Otago, Christchurch, 8011 New Zealand; 5 School of Life and Health Sciences, The Chinese University of Hong Kong (Shenzhen), Shenzhen, 518172 Guangdong China; 6 Department of Dermatology, the Second Affiliated Hospital of Dalian Medical University, Dalian, 116000 Liaoning China

**Keywords:** Nanomedicine, Nanoparticle, Cartilage, Bone, Regenerative medicine

## Abstract

With the gradual demographic shift toward an aging and obese society, an increasing number of patients are suffering from bone and cartilage injuries. However, conventional therapies are hindered by the defects of materials, failing to adequately stimulate the necessary cellular response to promote sufficient cartilage regeneration, bone remodeling and osseointegration. In recent years, the rapid development of nanomedicine has initiated a revolution in orthopedics, especially in tissue engineering and regenerative medicine, due to their capacity to effectively stimulate cellular responses on a nanoscale with enhanced drug loading efficiency, targeted capability, increased mechanical properties and improved uptake rate, resulting in an improved therapeutic effect. Therefore, a comprehensive review of advancements in nanomedicine for bone and cartilage diseases is timely and beneficial. This review firstly summarized the wide range of existing nanotechnology applications in the medical field. The progressive development of nano delivery systems in nanomedicine, including nanoparticles and biomimetic techniques, which are lacking in the current literature, is further described. More importantly, we also highlighted the research advancements of nanomedicine in bone and cartilage repair using the latest preclinical and clinical examples, and further discussed the research directions of nano-therapies in future clinical practice.

**Graphical Abstract:**

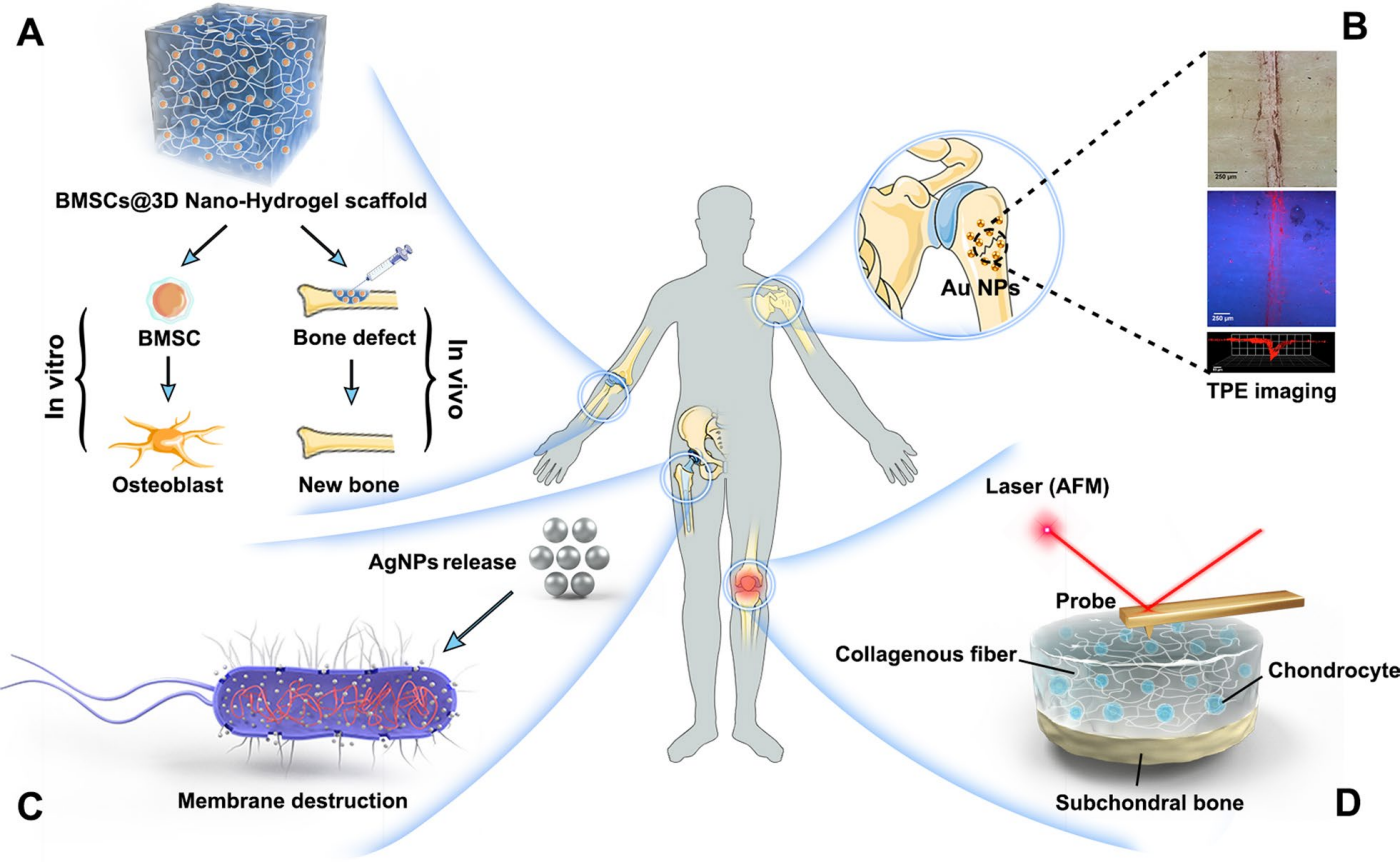

## Introduction

Nanomedicine combines nanoscience and medicine, aiming to understand the process and mechanism of biological activities from a microscopic level and apply nanotechnology to solve medical problems. Since most biomolecules exist and function at the nanoscale (1–100 nm), nanomaterials can impact cellular behaviours, which further regulates organ functions, playing a role in preventing and treating disease [1]. With the gradual improvement of theoretical knowledge in physics, chemistry and other basic disciplines, together with the continuous progression of analytic technology and fabrication process, nanotechnology has gradually revolutionized the medical field [2]. To date, the scope of nanomedicine has expanded into a multidisciplinary complex of research and applications, including but not limited to molecular diagnostics, in vivo imaging, targeted delivery and regenerative medicine [3].

Due to the demographic shift to an aging and obese society, the risk of cartilage [4, 5] and bone [6] damage in the population has increased significantly. Taking arthritis as an example, it is prevalent in clinical practice and induced by trauma, inflammation, or degeneration due to aging. The symptoms often include pain, dysfunction, and even joint deformity, overwhelming the healthcare system and impacting the living quality of patients [7]. Conventional surgical approaches such as microfracture and mosaicplasty, can alleviate pain and improve joint function; however, the new cartilage regenerated by these methods usually constitutes type I collagen and lacks type II collagen that has been observed in hyaline cartilage, resulting in unsatisfactory long-term clinical outcomes [8]. On the other hand, autologous/allogeneic bone grafting fails to avoid the lesions of the donor site with high risks of infection [9]. In addition, total joint replacement (TJR) suffers from wear (causing debris which is associated with aseptic loosening) and corrosion of the prosthesis, and is usually applied to the elderly patients at terminal stage [10]. Therefore, repairing cartilage and underlying subchondral bone has always been one of the major clinical challenges in orthopedics.

The utilization of nanomedicine in cartilage and bone repair may offer improved therapeutic effects by replacing diseased osteochondral tissue with native biological tissue, potentially restoring joint function. For instance, nanoparticle-based targeted labelling technology can rapidly assess bone quality and identify early cartilage defects that cannot be easily detected using existing monitoring approaches [11]. In addition, advances in nanomaterials have led to the development of a series of biomimetic scaffolds with nanostructures that enhance cell proliferation/migration and homing effects by simulating the natural bone hierarchy and extracellular matrix (ECM), to promote the regeneration of injured tissues [12]. Moreover, nanoparticles (NPs) and extracellular vesicles (EVs), as drug delivery vehicles, can minimize the dosage, increase the half-time and have the capacity to be modified, achieving targeted therapy for various types of osteochondral lesions [13]. Hence, the development of nanomedicine has significantly enhanced the diagnostics and therapeutics for cartilage and bone disease. Although recent literature has provided a holistic picture of nanotechnology applications in orthopedics, systemic reviews that comprehensively summarize different nano-based technologies, delivery modes, as well as the clinical translation of nanomedicine involved in cartilage and bone repair, remain elusive. Therefore, this review first describes current nanotechnologies in the medical field, followed by a systematic review of the development of nanocarriers and delivery modes in nanomedicine for improved delivery efficiency. The applications of nanotechnology in bone and cartilage repair are further discussed to provide an overall picture of nano-regenerative medicine. We believe that with continuous research, nanomedicine will play an increasingly important role in the prevention, diagnosis and treatment of cartilage and bone diseases.

## Medical applications of nanomedicine

The commonly accepted definition of nanomaterials refers to “A natural, incidental or manufactured material containing particles, in an unbound state or as an aggregate or as an agglomerate and where, for 50% or more of the particles in the number size distribution, one or more external dimensions is in size range 1–100 nm” [14]. Due to their nanoscale dimensions, substances have been removed from the realm of classical mechanics and replaced by the laws of quantum mechanics, resulting in unique properties in terms of melting point, conductivity, strength [15]. In addition, as the size decreases, the ratio of surface-to-volume increases dramatically, allowing for enhanced interaction between nanomaterials and biological systems, leading to improved functions.

### Nano-diagnostics

**Fig. 1:**
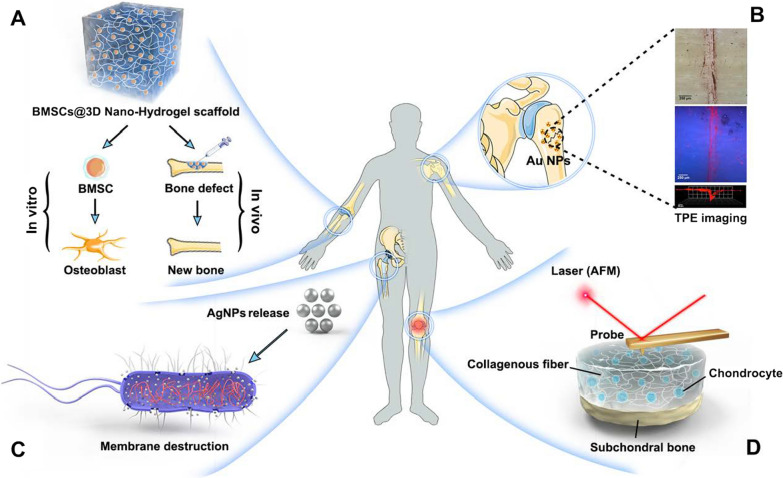
Medical applications of nanomedicine in orthopedics. **A** Novel biomimetic tissue repair nanomaterials. **B** Nanoparticulate MRI contrast agents create possibilities for low-radiation and high-resolution imaging of skeletal diseases. **C** Design and preparation of drug delivery vehicles with targeted functions using nanotechnology. **D** Application of AFM in the early detection and nanomechanical analysis of degenerative joint disease

Nano-diagnostics typically involve using nanomaterials for labelling, tracing, detection, signal enhancement or conversion in living organisms, and detecting biologically active molecules to achieve rapid diagnosis and point⁃of⁃care testing (POCT) of early diseases. The main research directions include bio-barcode assay (BCA) [16], nanofluidic array (biochip) [17], nanoparticles [18], quantum dot (QD) [19], and nanobiosensors [20], which are applied to ultra-sensitive detection, high-throughput and multiplex analysis, non-invasive cell tracking and cell dissociation, fluorescent labelling, and nanoprobe technology, respectively. For example, Borse et al. developed a lateral flow immunoassay (LFIA) method based on a double-antibody sandwich technique using fluorescent cadmium telluride QDs to detect inflammatory biomarkers including C-reactive protein and interleukin-6 (IL-6). The comparison of measurements with standard enzyme-linked immunosorbent assay (ELISA) showed that LFIA had good accuracy as well as sensitivity, which would be helpful in assessing the status of the implants [21]. In another study, Jin et al. developed a nitric oxide (namely NO, which is considered a biomarker of osteoarthritis) nanosensor for non-invasive and real-time assessment of osteoarthritis (OA) development. This nanosensor was synthesized by encapsulating the NO sensing molecules (i.e., 4-amino-5-methylamino-2',7'-difluorofluorescein Diaminofluorescein-FM) within the biodegradable poly (lactic-co-glycolic acid) nanoparticles. In vitro studies showed a positive correlation between the increase of fluorescence intensity and the change of NO concentration in chondrocytes. In vivo experiments confirmed its effectiveness to quantify the NO levels in joint fluid of a rat OA model [22]. Moreover, a gold nanoparticle-based biochip has been developed to detect osteoproteogerin (a protein indicative of osteoporosis), which can assess bone remodelling and provide accurate diagnosis of bone damage [23]. In addition, new technologies, such as atomic force microscopy (AFM), have been applied to analyze the micromechanical characteristics of bone tissue. Hengsberger et al. used a combination of AFM and nanoindentation techniques on trabecular and compact bone tissue to verify the advantages of AFM over conventional optical microscopy. They randomly selected four bone structural units (BSU) from dehydrated bone tissue and tested them with 24 indents at a maximum force of 5 mN. The results showed that AFM could effectively capture the surface features of BSUs and precisely locate the indentation areas and determine the intrinsic mechanical properties of each BSU, which were unmatched by conventional optical microscopy [24]. Therefore, AFM would be a potential solution for early detection and nanomechanical analysis of aging articular cartilage, providing valuable insights into the interaction between chondrocytes external cellular mechanical signals, and the regulation of chondrogenesis under normal or pathological conditions, through high-resolution imaging and real-time dynamic observation of living cells in culture (Fig. 1D).

### Molecular imaging

Molecular imaging is a popular direction in medical imaging research in recent years, aiming at the qualitative and quantitative study of biological processes at the cellular and molecular levels via imaging methods in the living state, thus enabling early, in vivo and targeted detection of diseases [25]. To date, the relatively mature technique uses isotope labelling imaging to detect precancerous lesions and microscopic tumours, which needs the injection of radioactive contrast agents with unsatisfactory image quality (e.g., positron emission computed tomography, PET). With the progression of nanotechnology, a large number of nanoparticulate magnetic resonance imaging (MRI) contrast agents (e.g., superparamagnetic iron oxide nanoprobes) have been developed, breaking the dominance of nuclear medicine agents used in molecular imaging and creating a possibility for low-radiation and high-resolution imaging of the skeletal diseases. Compared to nuclear medicine, MRI possesses higher spatial resolution (up to 25–100 μm) and enables multi-series imaging with simultaneous acquisition of anatomical and physiological information, which is yet to be seen in other imaging techniques [26]. For instance, Surender et al. synthesized europium-emitting surface-modified gold nanoparticles as MRI contrast agents and demonstrated their automatic enrichment on calcium ion-rich surfaces (fatigue-induced bone microdamage) for targeted labelling of microcracks within the bone (Fig. 1B). This technique can be used to detect bone mass and bone quality to identify the most likely locations of fractures for targeted early intervention, and the addition of gold nanoparticles may also reduce the concentration of contrast agents required for injection thereby lowering the toxicity of the probe [11].

### Nanoscale drug carriers

Nanoscale drug carriers refer to nanoparticles that use polymers (e.g., dendrimers [27]) or inorganic materials (e.g., quantum dots [28], metallic nanoparticles [29]) as carrier materials. Different nanocarriers may use different fabrication processes to combine raw drugs with carriers in a non-covalent or covalent binding manner. More importantly, they could be designed with targeting capability, stimulus responsiveness, biocompatibility, degradability, controllable release potential, together with some other unique properties to achieve an optimal therapeutic effect and reduced side effects (Fig. 1C). The main challenge in nanoparticle technology comes from the rapid clearance by the reticuloendothelial system (RES) in vivo, and the dominant countermeasure currently is to obtain invisible nanocarriers by coating their surface with hydrophilic polymers (e.g. polyethylene glycol, PEG), resulting in prolonging their persistence in the bloodstream [30]. Another approach to minimizing the non-specific clearance of nanoparticles is to wrap them in a cell membrane-derived biomimetic coating. These cell-like nanoparticles inherit the biological characteristics of the parent cells, with natural membranes on their surface capable of evading the immune system (immune escape), while having the ability to bind to specific cells due to the presence of membrane proteins (active targeting) [31]. In addition, the ability of extracellular vesicles to transport biomolecules to recipient cells holds great potential for drug delivery and promises, which can be considered as an alternative strategy to bypass the challenges of designing and manufacturing complex nanoparticles. This part will be discussed in detail in the following section.

### Nano-regenerative medicine

Novel biomimetic tissue repair nanomaterials and functionalized traditional materials have been developed for regenerative medicine (Fig. 1A). For example, nanofibers have a larger surface area as compared to conventional scaffold materials and thus can adsorb more proteins to provide more adhesion sites for cell membrane receptors, while the integration of nanoparticles into conventional biomaterials can enhance the mechanical properties as well as the biocompatibility of orthopedic implants, which is beneficial for osseointegration at the bone-implant interface [32]. In addition, the nanoscale characteristics of materials can significantly influence cellular behaviour, including cell adhesion, proliferation, differentiation, migration, and expression of different genes, which will be discussed in detail in subsequent sections.

## The delivery vehicle of nanomedicine

The ideal treatment should be the precise drug delivery to lesion sites with controlled-release so as to improve the pharmacokinetics and biodistribution of drugs and minimize the impact on non-target tissues. Nano drug delivery systems (NDDSs) are considered highly efficient targeted therapeutic carriers. Taking advantage of their high surface-to-volume ratio, drug loading capacity and stability, NDDSs can be distributed and retained in target tissues to exert therapeutic effect via the binding of ligands to specific receptors expressed on the surface of target cells, resulting in the reduction of the dose administered and alleviated side effects on the premise of ensuring drug efficacy, overcoming the limitations of conventional drug delivery methods [33, 34]. Often, nanoparticles and extracellular vesicles are two of the most widely studied nanocarriers.

### Nanoparticles

Nanoparticles (NPs) are microscopic particles manufactured from inorganic or organic materials up to 100 nm in size [13], which can be modified based on the specific requirement to achieve targeting delivery, stimulus responsiveness, and enhanced biocompatibility, optimizing their potential as targeted therapeutic carriers.

#### Inorganic nanoparticles

##### Metal-based NPs

Among numerous metal-based nanoparticles, gold nanoparticles (AuNPs) are the most intensively studied and have been made into various shapes (e.g., nanospheres, nanorods, nanoshells) due to simplicity in modification. AuNPs have great biocompatibility, and the *D*_*core*_ (the diameter of the gold core) > 15 nm of AuNPs have been proven to be comparatively nontoxic [35]. In addition to surface effect, quantum size effect and other substrate properties common to NPs, AuNPs also have some unique physical and optical properties, such as surface plasmon resonance (SPR) [36], which allows them to absorb light energy into heat, disrupting cell membrane permeability and protein physicochemical properties. This feature has been used in photothermal therapy to selectively damage specified cells to treat cancer [37]. Moreover, sensing systems based on AuNPs are emerging as a potential biomarker for the early diagnosis of degenerative joint diseases. It is well known that a disintegrin and metalloproteinase with thrombospondin motif-4 (ADAMTS-4) plays a critical role in the degradation of aggrecan (ACAN), and the detection of ADAMTS-4 activity in biological samples is of great significance for the screening of OA. Peng et al. developed a fluorescent turn-on AuNP probe to detect ADAMTS-4 activity in the synovial fluid of 11 knee surgery patients. The results showed a significantly higher fluorescence intensity in the acute joint injury group than that of chronic joint injury and end-stage OA groups, indicating high sensitivity and specificity of the AuNP-based probe [38].

Apart from gold nanoparticles, silver nanoparticles (AgNPs) are another popular materials that have been widely used. Silver is chemically stable with favourable electrical conductivity and broad-spectrum antibacterial properties. Metallic silver can damage the cell wall of bacteria and form coordination bonds with electron-donating groups to inhibit enzymatic activity, ultimately leading to bacterial death, while AgNPs can create stronger interactions with the bacterial surface, which in turn leads to a significant increase in bacterial inhibition performance [39]. Unlike antibiotics, the effects of AgNPs are not limited to a single mechanism but undergo multiple events simultaneously, with Ag^+^ release and reactive oxide species (ROS) production being the two most prominent types. The former can bind with the bacterial cell membrane to change the membrane potential and affect the normal physiological activities of bacteria, while the latter can directly or indirectly damage the cell structure, thus achieving the effect of inhibiting bacteria [40, 41]. Nowadays, AgNPs are often used for surface modification of certain special implants to reduce the incidence of periprosthetic infections. For example, in a study conducted by Gosheger et al., the femoral diaphysis of 30 rabbits was replaced with either a titanium prosthesis or an AgNPs-coated prosthesis. The prostheses were then manually contaminated with Staphylococcus aureus. As a result, the infection rate was significantly lower in the AgNPs-coated prosthesis group compared to the titanium-coated prosthesis group [42].

##### Magnetic NPs

Magnetic nanoparticles (MNPs) are highly effective reagents integrating diagnostics and therapeutics with a core of magnetically oriented ferrites (e.g., Fe_3_O_4_ and Fe_2_O_3_), which can be surface functionalized to improve its dispersibility and biocompatibility in aqueous solution [43–45]. More importantly, MNPs can deliver drugs or genes to the target area and maintain local release to exert therapeutic effects in the presence of an applied magnetic field, i.e. magnetic drug targeting (MDT), thereby reducing drug doses and keeping side effects to a minimum [46, 47]. For example, diclofenac sodium is a non-steroidal anti-inflammatory drug (NSAID) commonly used in clinical practice to treat OA, which requires high doses in administration to bring out sufficient therapeutic response due to its short half-life. In order to reduce the severe side effects resulting from frequent dosing, Arias et al. developed a diclofenac sodium-loaded MNP with iron as the core and ethylcellulose as the shell. This technique had an entrapment efficiency of about 54%, a sustained drug release time of up to 48 h, and a very suitable response to weak magnetic fields, holding good promise for use in degenerative joint diseases [48]. Furthermore, MNPs have been demonstrated to have the potential of inducing chondrogenic differentiation. Specifically, Son et al. isolated uniformly sized MNPs from Magnetospirillum sp. AMB-1 for the delivery and pelleting of bone marrow mesenchymal stem cells (BMSCs), and then simulated the three-dimensional (3D)-driven to which cells are subjected during limb development by applying a static magnetic field and/or magnet-derived shear stress. After 3 weeks of culture, the results showed that magnetic-force-induced biophysical stimulation substantially facilitated the synthesis of cartilage-specific ECMs (sulfated glycosaminoglycan, sGAG) and the expression of the chondrogenic gene (Col2A1) without affecting the hypertrophic differentiation of BMSCs [49]. Usually, the iron content of the nanocarrier used in the whole course of treatment does not exceed the total amount of conventional iron supplementation for patients with anemia, and the remaining magnetic particles can be safely excreted through skin, bile or kidneys [50]. Hence, the use of iron MNP is generally safe for human patients.

In addition to the above-mentioned iron-based MNPs being developed for the treatment of osteochondral injuries, super paramagnetic iron oxides (SPIOs) have been shown to label stem cells as a negative contrast agent (after being metabolized, the SPIOs contrast agent can increase the magnetic susceptibility of cells), which enables imaging of the seed cells survival in cartilage grafts, showing prospective applications for MRI monitoring of MSC-based OA ameliorating therapies [51].

##### Carbon-based NPs

Carbon-based NP, as compared to other nanoparticles, can be easily functionalized by bio-molecules via various surface coating strategies [52]. Therefore, they are a popular choice in biomedical applications. Common carbon-based NPs include carbon nanotubes (CNTs), graphene, fullerenes, nanodiamonds (NDs), and carbon dots (CDs). Among them, CNTs are considered to be one of the most promising materials for nano-delivery systems because of their cylindrical shape and nanoscale dimensions [53]. CNTs are the curls of graphene sheets and can be classified into single-, double- or multi-walled CNTs based on the graphene layer [54]. Due to their high flexibility, low mass density and high surface-to-volume ratio, CNTs possess high drug-loading capacity with excellent thermal, electrical and mechanical properties, and have been applied in biosensors and tissue engineering [55–57]. For example, it has been investigated that CNTs can act as bone substitutes when filled with calcium and arranged into the bone structure [58]. In another example, Li et al. demonstrated that multi-walled carbon nanotubes (MWCNTs) could concentrate more proteins and induce the expression of alkaline phosphate (ALP), cbfa1 and COLIA1 genes than graphite compacts, thereby promoting osteogenic differentiation of human adipose-derived mesenchymal stem cells (AMSCs) in vitro, which indicated their ability to modulate downstream stem cell responses without the addition of exogenous growth factors [59].

##### Silicon-based NPs

Among currently developed nanocarriers, mesoporous materials are a promising candidate, especially mesoporous silica nanoparticles (MSNs), whose high specific surface area and biocompatibility allow the delivery of various pharmacologically active molecules in a sustainable manner. The mesoporous structure and surface activity of silica can be modulated by altering additives in the preparation process and functionalized modifications, resulting in improved targeted delivery and controlled release [60]. For instance, Pasqua et al. developed an alendronate (ALN)-anchored MSNs drug-delivery system, with ibuprofen as the model drug for delivery and hydroxyapatite (HA) to mimic the bone matrix. The usage of ALN allows MSNs to obtain bone-specific drug delivery (ALN is electrostatically bonded to the external carboxyl functions of mesoporous silica on one side, and interacts with the surface of HA pellet on the other side). The results of biological tests confirmed their high biocompatibility and lack of off-target effects as well as the absence of any toxicity to normal cells [61].

##### Calcium phosphate-based NPs

Calcium phosphate (CaP) materials are highly similar to the mineral composition of bone tissue with excellent biocompatibility and osteoconductivity, which have been widely used in bone tissue engineering [62, 63]. In recent years, researchers have found that CaP NPs possess controllable particle size [64], high specific surface area [65], mild preparation conditions [64] and pH responsiveness [66], allowing most drugs to be adsorbed via ionic crosslinking or hydrogen bonding [67, 68], with the potential as delivery systems for bone repair therapy. For example, Zhou et al. prepared HA nanospheres with high specific surface area, which significantly improved the loading efficiency of bone morphogenetic protein-2 (BMP-2) and effectively reduced its initial burst release. They demonstrated that the loading capacity of nanospheres [80–150 nm] was significantly higher than that of microspheres (75–100 μm) [69]. In another study, Wang et al. loaded BMP-2 on biphasic calcium phosphate (BCP) NPs to obtain sustained release for up to 35 days and achieved excellent osteogenesis in a rat cranial critical-sized bone defects model. Impressively, the introduction of BMP-2 loading also improved the degradation efficiency of BCP and enhanced its biocompatibility [70]. Similarly, growth factors represented by vascular endothelial growth factor (VEGF) were also loaded on CaP NPs to promote bone defect repair and local vascular regeneration [71–73].

Taken together, the inorganic nanoparticles have the advantages of good mechanical properties, high thermal stability and wear resistance. However, they also have the shortcomings of difficult degradation, poor biocompatibility and low bioactivity, limiting their broader usage in downstream clinical applications.

#### Organic nanoparticles

##### Liposomes

Liposomes are composed of lipids such as phospholipids and cholesterol, which are structurally similar to biological membranes, leading to the spontaneous formation of closed vesicles in water. Their amphiphilic nature allows them to load with hydrophobic and hydrophilic drugs with improved cell uptake potential. More importantly, because of their structural similarity to the cellular membrane, liposomes have low immunogenicity [74]. Surface modifications (such as attachment of targeting ligands or coating with inert polymers) can further increase the circulation time and targeting activity of liposomes [75]. For instance, mannose incorporated liposomal delivery system (ML) was applied to deliver p-coumaric acid (CA), which was effectively targeting the inflammation site with increased sustain drug release, resulting in the inhibition of osteolytic differentiation of synovial macrophages in adjuvant-induced arthritic (AIA) rats by downregulating nuclear factor of activated T-cells c1 (NFATc1) expression [76].

##### Dendrimers

Dendrimers are hyperbranched polymers resembling branches of a tree. Their outer active functional groups can couple with biomolecules to improve their solubility and bioavailability, while effectively loading drugs into the core [77]. Until now, a large variety of cargoes, including diagnostic and therapeutic agents, have been loaded into dendrimers, and nucleic acids, together with small molecule drugs are the two most popular cargoes. For example, Zhong et al. investigated the effect of PEGylated polyamidoamine (PAMAM) dendrimer nanocarriers on the clinical pharmacokinetics and efficacy of OA modifier (insulin-like growth factor 1, IGF-1) through a surgical model of OA in rats. As a result, dendrimer-IGF-1 increased its residence time in rat knees tenfold compared to single injections of free IGF-1, and the efficacy of which at 4 weeks postoperatively was also significantly improved (60% reduction in the width of cartilage degeneration together with 80% reduction in the volumetric burden of osteophyte). This was attributed to PEG altering the surface charge of PAMAM dendrimers (making it positively charged) and subsequently enhancing the targeted transport of these cationic nanoparticles via electrostatic interactions with the negative charge of cartilage ECM, improving tissue binding, penetration, and GFs retention within joint cartilage [78].

##### Polymeric micelles

Polymeric micelles are core–shell structures formed by the self-assembly of amphiphilic block copolymers in water. The hydrophobic core and the hydrophilic shell of micelles are responsible for encapsulating hydrophobic drugs and maintaining the stability of micelle to avoid the exclusionary effect from RES in vivo, respectively [79]. For example, the chemotherapeutic drug (docetaxel)-laden micelles possessed high loading efficiency with a superior affinity for HA, resulting in enhanced in vivo antitumor activity, as well as the reduction in healthy bone tissue damage and systemic side effects. Therefore, this system had the potential to treat metastatic bone tumours [80]. In another example, Ye et al. developed doxorubicin (DOX)-loaded bone-targeting micelle using ALN as the target ligand, dextran (DEX) as the hydrophilic group, and 1,2-distearoyl-sn-glycero-3-phosphoethanolamine (DSPE) as the hydrophobic group. In vivo experiments showed that this micelle could deliver DOX to lung cancer bone metastasis sites and significantly prolong its retention time, inhibiting the growth of bone metastases tumours in the absence of systemic toxicity [81].

**Fig. 2:**
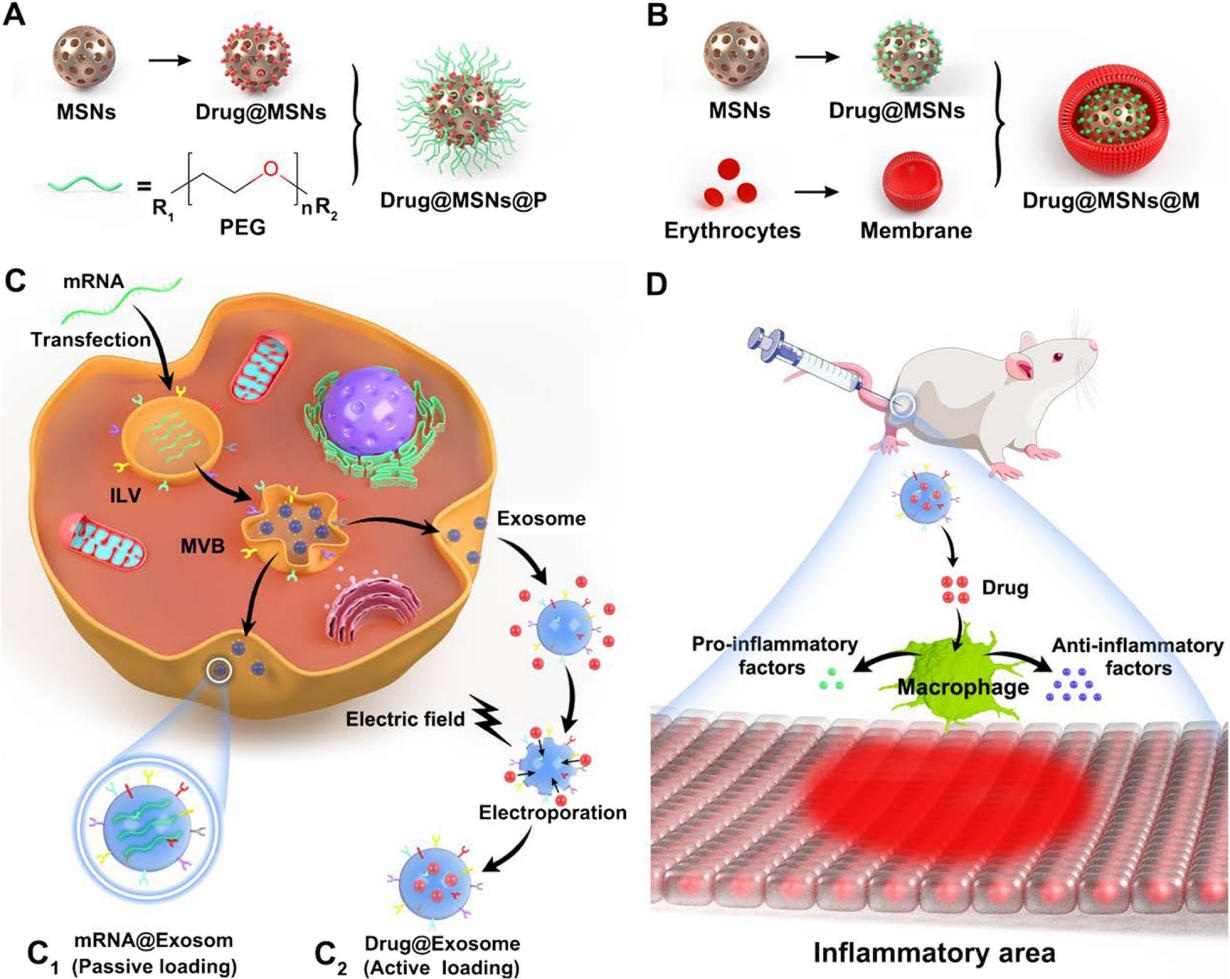
The delivery vehicle of nanomedicine. **A** Invisible nanocarriers can be obtained by coating NPs with PEG so as to bypass the immune system. **B** Cell-like NPs can be obtained by wrapping NPs in a cell membrane-derived biomimetic coating. **C**_**1**_ Indirect drug-loading: parent cells are transfected with specific mRNAs, leading to the release of exosomes overexpressing specific genetic substances. **C**_**2**_ Direct drug-loading: upon release from the parent cell, a high intensity electric field is used to form transient pores on lipid membranes, thereby facilitating the migration of therapeutic molecules into exosome. **D** Bone targeted delivery of Dex using exosomes increases its accumulation at the site of inflammation, inhibits pro-inflammatory cytokines and promotes anti-inflammatory cytokines, thereby significantly reducing joint inflammation

Overall, organic NPs have favourable biocompatibility and degradability. However, ordinary NPs are easily cleared in vivo by RES as foreign bodies. Therefore, surface modification with inert, biocompatible polymers is required to bypass the immune system, through reducing the uptake by macrophages, and decreasing distribution in the liver, spleen or lungs, resulting in prolonged in vivo circulation. PEG is one of the most commonly used stealth coatings to modify NPs through simple organic synthesis (Fig. 2A). The hydrophilicity and low antigenicity of PEG can promote the effective internalization of NPs by cells via fluid-phase endocytosis, and simultaneously resist protein adsorption, thereby increasing the spatial stability and biocompatibility of NPs [82]. In addition, multifunctional NPs can be synthesized in one step by mixing several polymeric building blocks. For example, Liang et al. constructed a stimuli-responsive NP with PEG and a poly (2-diisopropylaminoethyl methacrylate) (PDPA) homopolymer that exhibited not only a shift from hydrophobic to hydrophilic but also pH-dependent drug release, which provided extensive design flexibility for the improvement of nano-delivery systems [83].

### Biomimetic nanomedicine

Although PEG has been used for surface modification of NPs to reduce clearance by RES [84], recent studies have shown that the PEG-modified nano-medications are still being rapidly cleared by the liver after repeated administration, a phenomenon known as accelerated blood clearance (ABC). In addition, it also causes a hypersensitivity called complement activation-related pseudo allergy (CARPA) [85], limiting its further clinical translation. As a result, numerous research efforts have been devoted to the development of biomimetic nano-systems that are more compatible with in vivo drug delivery. Among them, the most prominent technologies are cell membrane-coated NPs and EV-based nanocarriers.

#### Cell membrane-coated NPs

Cell membrane-coated NPs are mainly realized by using natural cell membranes as a shell to encapsulate the synthetic NP cores (Fig. 2B). This method not only integrates various advantages of molecular proteins on the surface of cell membranes with the chemical properties of membrane materials, which gives NPs favourable biocompatibility and makes them camouflage as autologous components, thus escaping clearance by the immune system, but also prolongs their circulation time in the blood system, i.e., enhances passive targeting. More importantly, membranes of various cell sources have homologous targeting ability for different foci, which greatly enhances the enrichment of NPs at the target area, thus improving therapeutic efficiency and reducing toxic side effects. Depending on the purpose of administration, cell membrane biomimetic technology can replicate different membrane properties, mimicking the immune evasion of red blood cells (RBCs) [86], platelet-mediated adhesion along with aggregation [87], and even the homotypic binding phenomenon observed among tumour cells [88]. Although the applications of this nano-system in bone and cartilage repair have not been widely explored yet, there are some attempts to treat inflammatory arthritis. For example, neutrophil membrane-coated NPs have been shown to exert significant anti-inflammatory effects in a mouse model of collagen-induced arthritis (CIA), suppressing synovial inflammation and ameliorating the severity of joint damage by neutralizing pro-arthritogenic cytokines [89]. In another study, He et al. developed platelet-mimetic nanoparticles (PNPs) for targeted drug delivery in rheumatoid arthritis (RA) by coating platelet membranes onto poly (lactic-co-glycolic acid) nanoparticles. The results showed that the in vivo accumulation of PNPs in CIA mouse joints and their binding to inflammatory endothelial cells in vitro were substantially enhanced, with significant anti-inflammatory effects exerted by the loaded model drug FK506 [90]. In addition, Shi et al. demonstrated that the fusion of TRAIL-anchored cell membranes onto drug-loaded polymeric cores (TU-NPs) increased the drug accumulation in inflammatory regions of joints, providing a broad-spectrum antibacterial strategy based on NPs for the treatment of RA [91].

Despite the advantages of cell membrane-based nano-systems in improving therapeutic effect, some challenges still persist when dealing with the complex internal environment of the human body, such as cytotoxicity and bioaccumulation, together with high clinical translation costs [46, 92]. As a result, clinical research is shifting toward developing a more biomimetic and natural drug delivery platform to overcome the challenges and limitations associated with current targeted therapeutic strategies.

#### EV-based nanocarriers

EVs are naturally occurring nanocarriers, which are a collective term for various vesicles with membrane structures released by cells. EVs are heterogeneous in size and composition depending on their cell origin and can be mainly classified as exosomes (30–150 nm), microvesicles (MVs, 100–1000 nm), and apoptotic bodies (1–5 µm) [8].

The structure of EVs is similar to that of liposomes, except for a more complex bilayer. With the protective phospholipid bilayer, EVs are able to penetrate natural barriers in vivo (such as the blood–brain barrier) to deliver lipids, proteins, nucleic acids and other contents obtained from parent cells to recipient cells, thus mediating the intercellular communication [93]. Inspired by this physiological behaviour, researchers realize the potential of EVs in drug delivery and have created various methods to load exogenous therapeutic agents into EVs for therapeutic purpose [94].

*Indirect drug-loading* utilizes the endogenous loading mechanism to internalize therapeutic agents during the biogenesis of EVs via parent cells, including two key methods. In the co-incubation approach, therapeutic molecules are co-incubated with parent cells, resulting in the enriched molecules within the EVs secreted by the parent cells. This method is particularly popular for small-molecule chemical drugs with low cytotoxicity. For instance, Wang et al. used paclitaxel (PTX) and DOX to co-incubate with human cancer cells (breast MCF7, ovarian A2780) and obtained exosomes with appreciable drug levels from the culture medium supernatant [95]. Apart from co-incubation, another means is transfection. Parent cells are transfected with specific nucleic acid, leading to the release of EVs overexpressing specific genetic substances (Fig. 2C_1_). Liu et al. effectively promoted osteoporotic porous titanium (Ti) alloy osseointegration by expressing high levels of miR-20a in small EVs derived from BMSCs to increase its migration and osteogenic differentiation capacity [96].

*Direct drug-loading*: Directly loading drugs into EVs is more efficient than indirect drug loading, given that it provides precise control over the cargo within EVs. Normally, passive co-incubation and active loading via electroporation are the two approaches. Similar to co-incubation for indirect drug loading, passive co-incubation involves the co-culture between molecular drugs and EVs. In a study conducted by Wei et al., DOX was loaded into exosomes isolated from BMSCs through co-culturing for 30 min, desalinizing with triethylamine at room temperature for 1 h and then dialyzing against phosphate-buffered saline (PBS) at 4 °C overnight. The experimental results showed that the prepared exosome-DOX exhibited high uptake efficiency of osteosarcoma MG63 cell line while low cytotoxicity in H9C2 (cardiomyociytes) cell line, which provided a promising chemotherapeutic strategy for targeted therapy of osteosarcoma [97]. The main principle of electroporation, on the other hand, is to use a high-intensity electric field to form transient pores on lipid membranes, which facilitates the migration of therapeutic molecules into EVs (Fig. 2C_2_). Faruqu et al. encapsulated small interfering ribonucleic acids (siRNA) into exosomes secreted by human embryonic kidney cells (HEK-293 cells) via electroporation, used for inhibiting the growths of human pancreatic adenocarcinoma (PANC-1) cancer cells [98].

Exosomes have high targeting potential due to membrane proteins, and are more suitable as candidate nanocarriers for targeted drug delivery because of their easier surface modification and lower immunogenicity than MVs and apoptotic bodies [99]. For example, it has been shown that the conjugation of BMSC-specific aptamers to bone marrow stromal cell (ST)-derived exosomes (STExos) could effectively enhance their targeting to bone (promoting osteogenic differentiation of BMSCs) while reducing their accumulation in the liver and lungs (avoiding the rapid metabolism and clearance), thereby increasing bone mass in an ovariectomy (OVX)-induced postmenopausal osteoporosis mouse model as well as accelerating bone healing in a femur fracture mouse model [100]. Similarly, Yan et al. established a folic acid (FA)-PEG-cholesterol (Chol) compound surface-modified exosome encapsulating dexamethasone Dex nanoparticle (FPC-Exo/Dex) to achieve targeted therapy for RA. Their result demonstrated that an increased accumulation of Dex at inflammation sites inhibited pro-inflammatory cytokines and promoted anti-inflammatory cytokines, resulting in a significant reduction of joint inflammation in CIA mice (Fig. 2D). Safety evaluation further demonstrated its good biocompatibility as well as low hepatotoxicity [101].

In addition, it has been shown that during bone remodelling, all types of bone cells can spontaneously secrete exosomes to mediate the cellular communication and crosstalk between osteoblasts (OBs) and osteoclasts (OCs), cooperatively maintaining bone homeostasis in vivo. As an example, exosomes secreted by OCs had been proved to inhibit osteoblast activity along with osteoblastic bone formation [102, 103], while exosomes derived from OBs [104] and BMSCs [105] played an important role in regulating osteoclast differentiation and osteoblast activity, respectively. Besides, Song et al. demonstrated that exosomes secreted by vascular endothelial cells (EC-Exos) exerted better bone targeting performance than OBs- or BMSCs-derived exosomes, which significantly inhibited osteoclastogenesis and osteoclast activation, as well as suppressed ovariectomy-induced secretion of pro-inflammatory cytokines, reducing bone resorption [106]. These findings provide a drug-free therapeutic strategy based on naturally occurring exosomes for the treatment of bone diseases.

Although exosome-based therapies have not yet gained clinical approval, risk assessments from early phase clinical trials generally support the safety of exosome administration [107]. From being used as cell garbage cans [108] to becoming prospective nanocarriers for various therapeutic agents, the mechanism of exosome-mediated intercellular communication is gradually being revealed [109]. Compared to cell membrane-coated biomimetic NPs, exosome-based NDDSs have the advantages of intrinsic cargo loading capacity, easier surface modification, high targeting potential, low immunogenicity, long circulation half-life together with efficient cellular internalization [110]. However, challenges such as isolation and purification, scale-up production, and proper exosome storage need to be addressed before the clinical translation [107]. In addition, the internalization and transport mechanisms of MVs and other EVs subpopulations in recipient cells need to be investigated further to facilitate the expansion of EVs applications in targeted therapeutic areas.

## Nano-medicine in bone repair

**Fig. 3:**
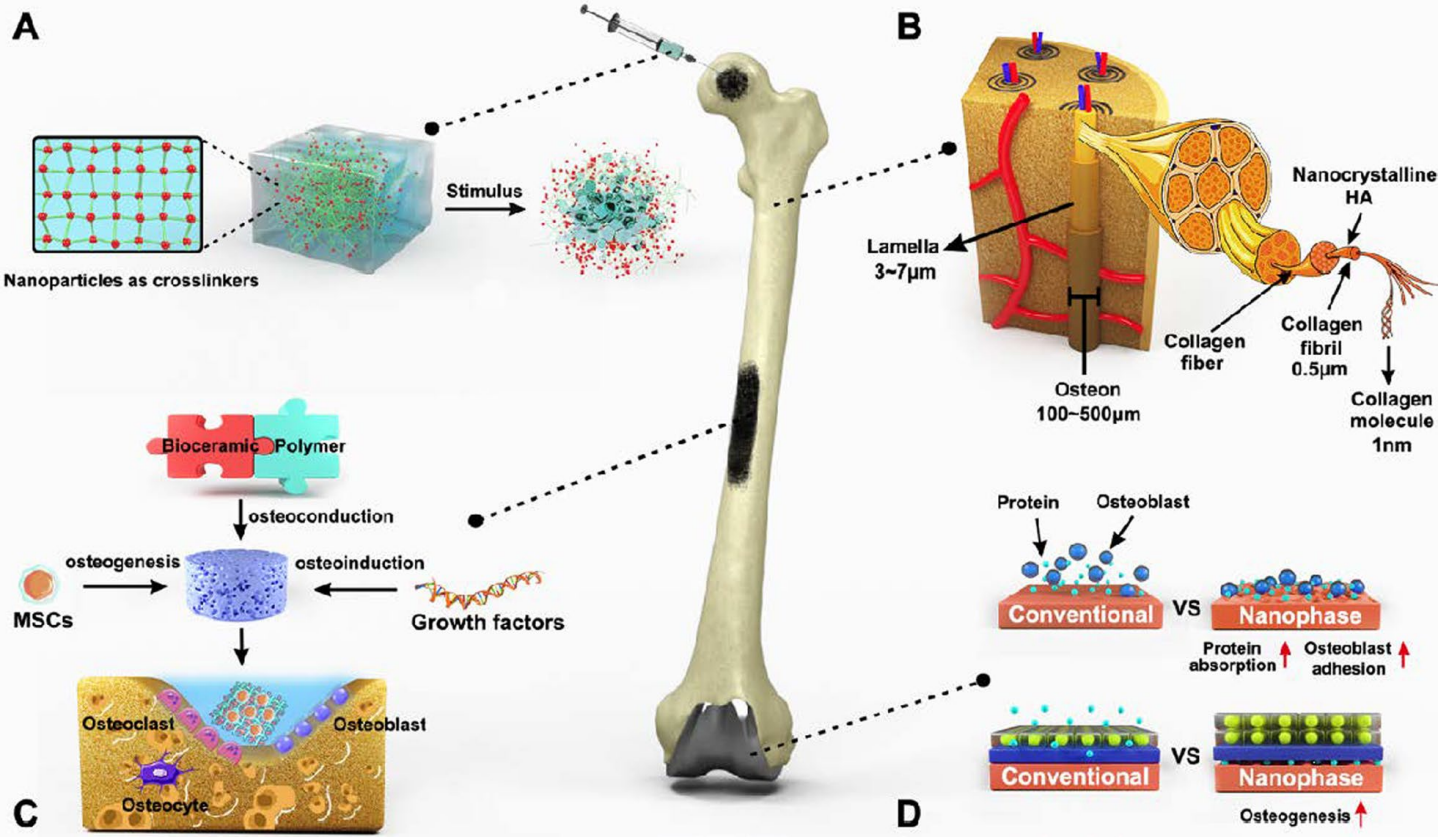
Nanotechnologies in bone repair. **A** The introduction of nanoparticles can effectively improve the structural properties of the hydrogel network with enhanced mechanical properties, while imparting stimulus responsiveness. **B** The ECM of bone tissues mainly consist of highly ordered collagen nanofibers and nanocrystalline HA. **C** Three essential elements of nanocomposite scaffold-mediated bone regeneration and the cellular composition of bone tissue. **D** Generating nano-surface features on metallic implants through surface modification to enhance the adsorption of proteins as well as adhesion of osteoblasts, thus promoting osteogenesis

Bone is a mineralized connective tissue, containing three types of cells (osteoclast, osteoblast and osteocyte) and a biphasic ECM (the content ratio of mineral part to organic part is approximately 7:3) [111]. The metabolism of bone starts with the formation of the skeleton by cells and collagen fibres (mainly COL-I). Minerals are further deposited into the skeleton, which is regulated by growth factors. Together, the minerals and skeleton contribute to bone tissue's high strength and toughness, thereby providing sufficient support and protection for normal physical activities [112]. Based on the distribution and porosity of blood vessels, bone can be divided into the cortical bone, which is overlaid on the outside with few blood vessels and low porosity (10%), and cancellous bone, which is the key component in the inner composition with abundant blood vessels and high porosity (50%–90%) [111]. Thanks to the relatively adequate blood supply, bone tissue has a certain regenerative potential and can renew itself via constantly undergoing bone remodelling in order to adapt to the ever-changing body load and maintain the necessary mechanical strength [113]. However, not all bone injuries can heal on their own. Once damage exceeds the maximal self-repair capacity of bone, external intervention is required [114]. Conventional bone repair strategies often cannot achieve rapid and effective regeneration of bone tissue due to their inherent shortcomings. For instance, autografts have limited donor availability and can cause donor site morbidity. Meanwhile, allografts are subject to a high risk of immune rejection and potential disease transmission [115]. Other metallic or non-metallic implants used to reconstruct the structure and function of bone tissues at the injured site also have to face the issues such as corrosion resistance and bacterial adhesion [116]. Therefore, the development of new bone repair materials is required. Since bone ECM mainly consists of highly ordered collagen nanofibers and nanocrystalline HA [117] (Fig. 3B), nanomaterials with biomimetic features and excellent physicochemical properties have gained popularity in orthopedic clinical trials and applications.

### Nano-biomaterials for bone grafts

Fractures, osteoporosis and OA due to injuries, tumours and the aging process are the most common causes of bone loss [118–120]. It is well documented that bone loss greater than two times the diameter of the long bone diaphysis (i.e., a critical bone defect) is unlikely to heal on its own, despite the advancement in clinical management [114]. Although autologous bone grafting is still considered as the gold standard for bone repair [121], insufficient bone supply and unavoidable donor site morbidity have significantly limited its application. In recent years, bone tissue engineering has developed rapidly for bone defect treatment, and has achieved considerable advancement. In particular, nano-scale biological scaffolds demonstrated improved capability to mimic the 3D structure of natural ECM, promoting the adhesion, proliferation and differentiation of osteoblasts, with the reduced limitations of traditional graft repair methods (Table 1).

**Table 1:** Examples of implantable nanomaterials used in bone repair

Material		Fabrication Technique	Cell type/Animal modle	Outcomes	Refs.
Single component	*PCL*	electrospinning	rMSCs	The electrospun PCL scaffolds provided an environment that supported mineralized tissue formation	[129]
		electrospinning	hOB cells	A 3D thick scaffold (93% porosity) was fabricated by changing the process parameters and PCL solution characteristics	[132]
		TISA	mBMSCs	The highly porous (96.4%) TISA scaffold acted as favourable synthetic ECM for functional bone regeneration through a physiological endochondral ossification process	[133]
Nanocomposites	*PCL/gelatin*	electrospinning	hMSCs	The combination of PCL and gelatin endowed the scaffold with both structural stability of PCL and bioactivity of gelatin, providing a structurally and biochemically improved 3D ECM-like microenvironment for cell infiltration and proliferation within the scaffold	[135]
	*PLLA/PCL*	EYA	hESC-MSCs	EYA technology made it possible to construct 3D scaffolds with good mechanical strength and sufficient interconnected micropores in a functionally graded structure	[131]
	*PLA/nβ-TCP*	freeze-dry	muscular pockets of rabbits	The nβ-TCP content significantly influenced the in vitro degradation and in vivo osteoconductive properties of the composite scaffolds	[136]
	*gelatin/β-TCP*	electrospinning	rBMSCs	The composite scaffolds promoted osteogenic differentiation of BMSCs in vitro and bone regeneration in vivo by activating Ca^2+^-sensing receptor signaling	[137]
	nTiO_2_/PLGA	sonication	hOB cells	The nTiO_2_/PLGA composites sonicated to have nanometer surface roughness values could improve osteoblast functions	[138]
	*HA–TSF*	coaxial electrospinning	MG-63 osteosarcoma cells	The nanocomposite had good biomimetic and mechanical properties and was more effective than pure silk in inducing cell adhesion, proliferation and bone formation	[148]
	*nHA/PLLA*	TIPS	none	The incorporation of nHA improved the mechanical properties and protein adsorption of the composite scaffolds while maintaining high porosity and suitable microarchitecture	[149]
	*GelMA-GNP*	photo-crosslinking	hADSCs	The hydrogels loaded with GNPs promoted proliferation, differentiation, and ALP activities of hADSCs as they differentiated towards osteoblast cells in dose-dependent manner	[154]
	*Sr-GelMA*	short vortex spinning	hMSCs	The addition of Sr nanoparticles greatly enhanced the printability of the composite bioink, and scaffolds bioprinted from it remained stable through 28 days of culture, showing vast MSCs osteogenic differentiation capacity	[156]
	*GelMA/MNPs*	photo-crosslinking	hMSCs, MC3T3s	The adjustable mechanical properties of hydrogels could be achieved by controlling the size and concentration of MNPs	[157]
	*PIC/MWCNTs*	ultrasonication	rBMSCs	The introduction of MWCNTs into the PIC hydrogel could stimulate the proliferation and osteogenic differentiation of BMSCs	[159]
	*PECE/Collagen/nHA*	ultrasonication	cranial defects of New Zealand White rabbits	The hydrogel composite had both injectability and thermo-sensitivity, and showed good capacity to guide bone regeneration, which had great potential in the minimally invasive repair of bone defects	[160]
	*Alginate/gelatin/SiO*_*2*_	chemical crosslinking	hUMSCs	Biocompatibility and osteogenic ability of the hydrogels were significantly increased with the addition of SiO_2_	[161]
	*GelMA-G-MBGN*	co-crosslinking	MC3T3-E1 cells	This enhanced organic − inorganic hydrogel membrane could maintain localized body fluid environment stability under the premise of promoting vascular regeneration to accelerate bone tissue reconstruction	[163]
	*PNAGA-Clay*	physical crosslinking	ROB	The hydrogen bonding of nanoclay contributed to the superior mechanical performances as well as swelling stability of the hydrogels	[164]
	*LPN-GelMA*	DW	hBMSCs	Developing a novel light-curable nanocomposite bioink for 3D skeletal regeneration supportive of cell growth and growth factor retention and delivery	[165]
	*CHPOA-PEGSH*	chemical crosslinking	mouse calvarial bone defect model	CHPOA/hydrogel was an efficient delivery system for coadministration of FGF18 and BMP2 with the potential to improve the ratio of complete healing of calvarial defects in individual mice	[166]

PCL, poly (ε-caprolactone); rMSCs, rat mesenchymal stem cells; hOB, human osteoblast; TISA, thermally induced self-agglomeration; mBMSCs, mouse bone marrow mesenchymal stem cells; hMSCs, human mesenchymal stem cells; ECM, extracellular matrix; PLLA, poly (L-lactic acid); EYA, electrospinning-based yarn assembly; hESC-MSCs, human embryonic stem cell-derived mesenchymal stem cells; PLA, poly (lactic acid); nβ-TCP, nano-sized β-tricalcium phosphate; nTiO_2_, nanophase titania; PLGA, poly- (lactide-co-glycolic); HA, hydroxyapatite; TSF, tussah silk fibroin; TIPS, thermally induced phase separation; GelMA, methacrylated gelatin; GNP, gold nanoparticles; hADSCs, human adipose-derived stem cells; ALP, alkaline phosphate; Sr, strontium-carbonate; MNPs, magnetic nanoparticles; MC3T3s, murine-derived preosteoblasts; PIC, polyion complex; MWCNTs, multiwalled carbon nanotubes; PECE, triblock copolymer poly (ethylene glycol)-poly (ε-caprolactone)-poly (ethylene glycol); hUMSCs, human umbilical cord mesenchymal stem cells; PNAGA, poly (N-acryloyl glycinamide); ROB, rat osteoblast; LPN, a synthetic nanoclay, Laponite®; DW, deionised water; CHPOA, acryloyl group-modified cholesterol-bearing pullulan; PEGSH, thiol group-modified polyethylene glycol; FGF18, recombinant human fibroblast growth factor 18; BMP2, recombinant human bone morphogenetic protein 2

Bone tissue engineering can be simplified into the loading of seed cells as a source of new bone formation, the incorporation of scaffolds to support cell adhesion and migration, and the addition of bioactive molecules that promote osteogenic differentiation, which is consistent with the essential elements of bone regeneration (i.e., osteogenesis, osteoconduction and osteoinduction) in human body [115] (Fig. 3C). The scaffold is the key framework to facilitate cell attachment and growth and acts as one of the ECM components, mediating intercellular signalling and interactions [122]. Thus, the capability of the scaffold to mimic the structure and composition of the bone dictates the success of tissue regeneration. An ideal bone tissue scaffold material needs to have good biocompatibility and biodegradability, suitable pore size and porosity, as well as certain mechanical properties. In addition, it should be able to control the release of bioactive molecules through surface modification, thus regulating the adhesion, proliferation and osteogenic differentiation of seed cells [123]. The advances in nano-biomaterials have made it possible to develop biomimetic scaffolds, which can possess a similar hierarchic organization of native bone, as compared to earlier scaffold materials (metals, ceramics and polymers), thereby efficiently mobilizing corresponding cells at the bone-graft interface during the bone remodelling while providing adequate mechanical properties to adapt to a variety of loading environments.

#### Nanofibers

Nanofibers (NFs) are more suitable for scaffold components than nanoparticles due to their continuous structure. The advantages of nanofibrous scaffolds include high porosity and surface-to-volume ratio, as well as a morphological similarity to natural ECM, which can reconstruct a biomimetic microenvironment to affect the cell–matrix interactions, producing favourable cell behaviours (adhesion, proliferation, differentiation) [124]. Common fabrication processes for NFs include electrospinning, phase separation and self-assembly, with electrospinning being the most popular, mainly because of its simplicity and the versatility of prepared scaffolds [125, 126]. In electrospinning, the polymeric solution is subjected to an electrostatic force (Maxwell stress) beyond its surface tension under applied voltage. Therefore, when the polymeric solution is ejected from charged jets, it undergoes multiple stretching and splitting and creates nanoscale fibres after the solvent evaporates [127]. To date, natural polymers, including collagen, gelatin, chitosan (CS) and alginate, and synthetic polymers such as poly (lactic acid) (PLA), poly (glutamic acid) (PGA) and poly (ε-caprolactone) (PCL) [128], have been used to fabricate NFs.

##### Single-component nanofibrous scaffolds

**Fig. 4:**
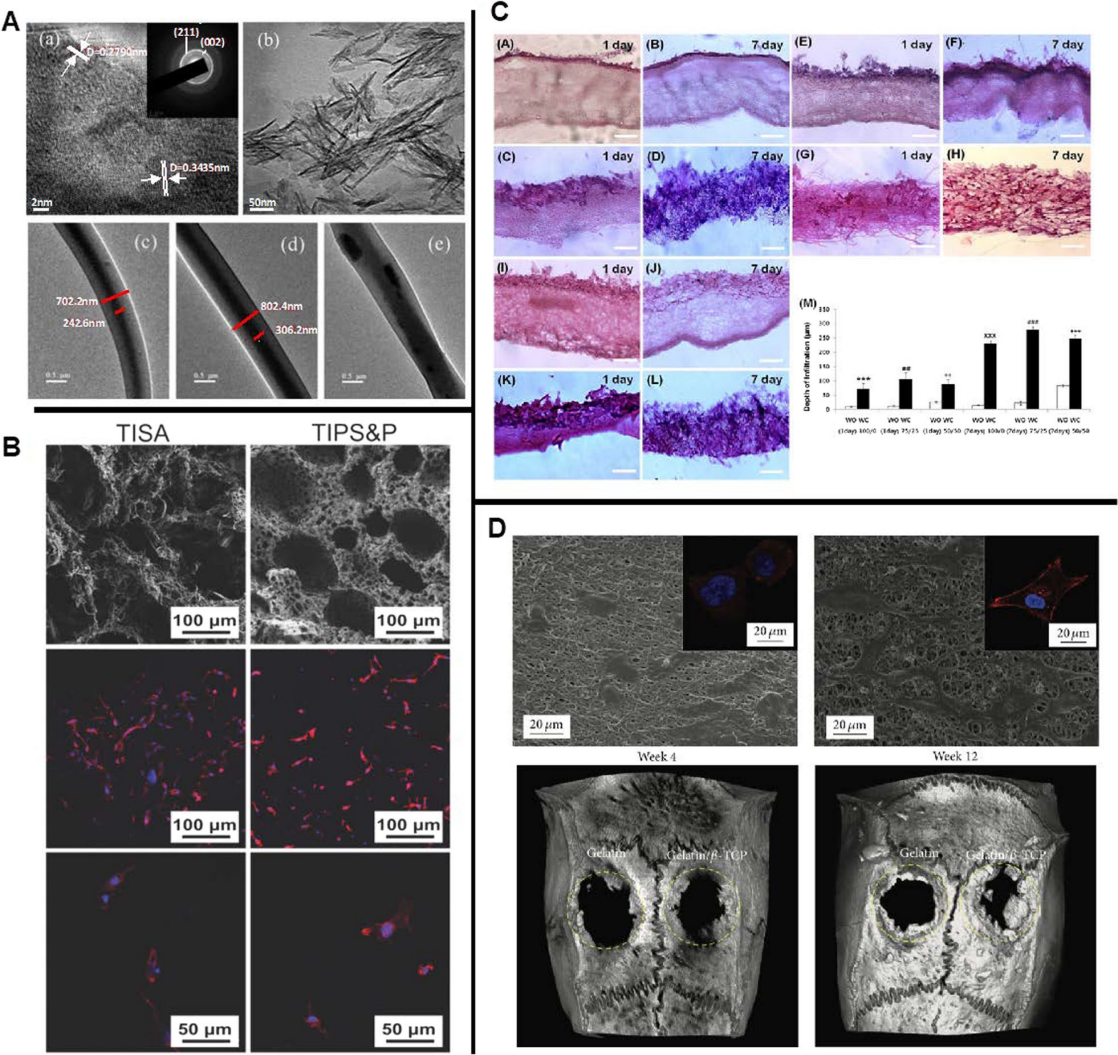
**A** The composite fibres fabricated with a core–shell design are distinctive, and a fuzzy boundary between the core and shell implies good miscibility [148]. **B** mBMSCs were able to attach and spread well on the constructs [133]. **C** H&E staining results indicated that the PCL/gelatin composite electrospun scaffolds with crater-like structures showed a significantly higher affinity for cell infiltration [135]. **D** Micro-CT analysis showed that the composite scaffold group had more bone in-growth than the pure gelatin group [137]

Interestingly, natural polymers are much less popular for bone repair despite their superior biocompatibility and enriched arginine-glycine-aspartate (RGD) components. This may be due to insufficient mechanical strength and uncontrollable in vivo degradation. Therefore, there is a growing interest in synthetic polymers with better mechanical properties and controllable degradation rates in bone tissue engineering. For example, Yoshimoto et al. found that the osteoblast-like cells were attached to the surface of electrospun PCL after 4 weeks of dynamic culture with rat MSCs. More importantly, satisfied COL-I deposition and matrix mineralization were oberserved [129], indicating the potential of electrospun PCL scaffold as a bone tissue scaffold. However, conventional electrospinning produces dense and compact NFs with small superficial pores. In addition, it can only fabricate two-dimensional (2D) scaffolds that are not conducive to cell penetration and signal transduction, reducing the long-term survival of transplanted cells [130, 131]. As a result, researchers have been making efforts to improve the fabrication process in order to optimize the porous structure (e.g., size, interconnectivity and porosity) of NFs. Eap et al. fabricated a 3D scaffold with a heterogeneous distribution of macropores and a porosity of 93% by changing process parameters (such as voltage, feed rate) and PCL solution characteristics (such as viscosity, conductivity). They demonstrated that a 3D scaffold could enhance cell proliferation in vitro compared to a 2D scaffold [132]. Another 3D PCL scaffold fabricated by Xu et al. using thermally induced self-agglomeration (TISA) technology could increase the porosity to 96.4% with a maximum pore size of 300 μm, which more closely resembled the natural ECM in terms of topography/morphology. In vitro studies showed that the scaffolds not only produced higher cell viability (Fig. 4B), but also promoted BMP-2-induced chondrogenic differentiation of mouse BMSCs, followed by functional bone regeneration through a physiological endochondral ossification (EO) process [133]. Apart from electrospinning, other techniques have also been developed for NFs fabrication. Hartgerink et al. adopted a pH-controlled self-assembly process to synthesize a peptide-amphiphile scaffold that allowed reversible cross-linking of NFs. The mineralization experiments showed that the peptide-amphiphile NFs could form well-aligned HA on their surface, which was highly consistent with the nanostructure of native bone [134].

##### Composite nanofibrous scaffolds

As compared to the improvement of single-material scaffolds, the general trend of current research is to design hybrid scaffolds made of different material combinations according to practical needs. Due to the synergistic effects among the components, which compensate for the major drawbacks of each material, the composites tend to exhibit better bioactivity and osteogenic capacity. For example, Hwang et al. developed a PCL/gelatin composite scaffold with a crater-like structure, which had comparable surface chemistry, degradation rate and mechanical properties as common scaffolds, with increased pore size and porosity [135]. The presence of gelatin provided the bioactivity that was lacking from PCL. Cells showed improved infiltration and proliferation in this hybrid scaffold (Fig. 4C). Furthermore, Cai et al. produced a 3D macroporous nanofibrous scaffold (MNF) from electrospun PCL and poly (L-lactic acid) (PLLA) through sequential yarns manufacture and honeycombing process (EYA technique), and investigated its therapeutic effect on the repair ability of defective bone tissue in a rabbit tibial model. The results showed that the composite scaffold exhibited good structural integrity by hybridizing PCL and PLLA. More importantly, the EYA technique made it possible to create functionally graded structures with porous characteristics at two additional orders of magnitude, which provided effective structural integrity support for MSC growth and guided bone formation [131].

In addition to the polymer/polymer scaffolds mentioned above, composite scaffolds fabricated by mixing nanoceramic particles with degradable polymeric solutions can also optimize the mechanical and biological properties of the NFs required for bone regeneration. Cao et al. manufactured NFs with nano-sized β-tricalcium phosphate (nβ-TCP) and PLA using a freeze-drying method. The fabricated scaffold showed increased osteoconductivity. More importantly, the composite scaffolds containing 30% nβ-TCP demonstrated enhanced mechanical properties, which was beneficial for bone regeneration [136]. In another study, Zhang et al. designed a composite scaffold composed of β-TCP nanoparticles and electrospun gelatin NFs to repair calvarial defects in a rat model. The result showed that the composite NFs group significantly increased ALP activity and osteogenic gene expression, inducing more extensive osteogenesis and neovascularization than the gelatin-only group (Fig. 4D) [137]. Also, in Liu’s work, nanophase titania was incorporated into poly- (lactide-co-glycolic) (PLGA) scaffolds via sonication technique, which made the composite degradable while mimicking the surface roughness of native bone, giving rise to an improvement in osteoblast functions (e.g. cell attachment, calcium-containing mineral deposition) [138].

Among numerous ceramic/polymer scaffolds, the combination of nano-hydroxyapatite (nHA)/polymer has been identified as an ideal scaffold in bone graft because it facilitates cell proliferation, differentiation, and ECM deposition. Furthermore, as the main inorganic component of native bone, the introduction of nHA can further mimic the natural bone microenvironment (specifically, nHA and polymers are assembled into mineralized fibres and 3D porous scaffold materials simulate the microstructure of cancellous bone), further enhancing the bone-remodelling ability of the scaffold. Therefore, it has been widely studied and used in bone tissue engineering [139–144]. The traditional preparation method of apatite–polymer composite induces apatite nucleation by modifying the polymer with functional groups, followed by immersion in simulated body fluid (SBF) with ion concentrations similar to those of human plasma, depositing bone-like apatite layer onto the polymer surface [145, 146]. For example, Thien et al. used this method to prepare HA/CS composite and revealed an approximately equal calcium/phosphorus ratio of HA deposited on the nanofiber surface similar to native bone. In addition, cells exhibited improved proliferation and osteogenic differentiation on HA/CS composite compared to pure CS NFs [147]. Unfortunately, the time-consuming fabrication process, together with the lack of nanostructure of HA within the scaffold to biomimic the bone microenvironment, impeded further utilization of HA/CS composite scaffold in clinical translation. With rapid technological development, nHA/polymer composite scaffolds have been successively developed. For instance, Shao et al. fabricated a nanostructured composite with HA nanoparticles as the core and tussah silk as the shell via coaxial electrospinning (Fig. 4A). They demonstrated that this composite fibre had an excellent biomimetic capability and mechanical properties to effectively support the proliferation of osteoblast-like MG-63 cells and promote the mineralization within the scaffold [148]. In another study, Wei et al. manufactured a novel nHA/PLLA composite scaffold using a thermally induced phase separation (TIPS) technique. They demonstrated that the introduction of nHA could improve the protein adsorption capacity and mechanical properties of the material while maintaining high porosity and suitable microarchitecture [149].

#### Hydrogel-based scaffolds

Hydrogels are physically and chemically cross-linked hydrophilic scaffolds that could share a high degree of similarity to ECM [150]. Often hydrogel is highly hydrated with low viscosity, and it can incorporate growth factors, drugs, or cells for tissue engineering and be injected directly into the defective bone site, avoiding the complexity of surgical or invasive implantation. However, hydrogels lack sufficient mechanical strength, resulting in unsatisfactory therapeutic effects [151]. In order to improve the mechanical properties of hydrogels, nanoparticles have been introduced (Fig. 3A). Nanoparticles have a large specific surface area, which can form a tight interface with the hydrogel structural chain, improving the mechanical properties of hydrogel. In addition, nanoparticles also possess good osteoconductivity, biocompatibility and biofunctionality to promote cell adhesion, proliferation and osteogenic differentiation [152]. Based on these advantages, nanocomposite hydrogels have good prospects for application in bone tissue engineering [153].

Many studies have investigated the modification of hydrogels using metallic nanoparticles or their oxides, and have confirmed that different metal ions impart distinct redox responsiveness to the materials. For example, methacrylated gelatin (GelMA) hydrogels loaded with AuNPs constructed by HEO et al. could promote the proliferation, differentiation and ALP activity of human AMSCs, leading to osteoblast differentiation due to the activation of the p38 mitogen-activated protein kinase (MAPK) pathway by AuNPs [154]. Whereas the calcium ions, in addition to significantly increasing the hardness of the hydrogel, also confer a shape memory function [155]. Alcala-Orozco et al. incorporated strontium nanocomposite into GelMA hydrogel to improve BMSC osteogenesis via simulating the extracellular signal-related kinase (ERK)1/2 signalling pathway [156]. Besides, Jaiswal et al. demonstrated that the adjustable mechanical properties of hydrogels could be achieved by controlling the size and concentration of MNPs [157].

In addition to the metallic nanoparticles, CNTs are another popular additional material for hydrogel based scaffolds, aiming to improve cells osteogenic differentiation in bone tissue engineering. For example, Zhang et al. added rosette nanotube (RNT) into poly (2-hydroxyethyl methacrylate) (pHEMA) hydrogel and modified their surface with RGDSK (Arg-Gly-Asp-Ser-Lys) to adsorb more fibronectin (a protein that promoted osteoblast adhesion) via lysine or RGD groups functionalized on RNTs, thereby increasing osteoblast density and facilitating bone tissue regeneration [158]. Cui et al. combined MWCNTs with a tough polyion complex (PIC) hydrogel to fabricate a nanocomposite hydrogel via 3D printing technology, aiming at providing a suitable environment for bone regeneration in vivo. The result indicated that this nanocomposite hydrogel had high mechanical strength and good biocompatibility. Encapsulated BMSCs showed enhanced osteogenic and vascular differentiation ability due to mineralized matrix formation and upregulation of osteogenesis-related genes. This conclusion was similarly validated in repairing a calvarial defect Sprague–Dawley (SD) rat model [159].

Apart from carbon-based nanoparticles, other inorganic non-metallic nanoparticles have also been used as nano-fillers to promote bone regeneration, such as CaP, which is similar in composition to the inorganic components of normal calcified tissues in the human body, contributing to the improvement of the biocompatibility and osteoconductivity in nanocomposite hydrogels. For instance, the thermal-responsive PEG-PCL-PEG copolymer (PECE)/Collagen/nHA hydrogel composite developed by Fu et al. showed an enhanced capacity to guide bone regeneration in vivo, which flowed freely at room temperature and lost flowability near body temperature, providing a novel strategy for minimally invasive repair of non-loading defects in orthopedic [160]. At the same time, silica nanoparticles are modified with carboxyl groups of hydrogels, including sodium alginate and gelatin, via hydroxyl functional groups on their surface, resulting in the enhanced mechanical strength and viscosity of hydrogels [161]. More importantly, the silica fraction released from them can also enhance the angiogenic ability of endothelial cells by increasing the gene expression of pro-angiogenic cytokine receptors and upregulating downstream signalling pathways [162]. Furthermore, co-cross-linked hydrogels formed by mesoporous bioactive glass nanoparticles (MBGNs) and GelMA exhibit durable degradation time and superior biomineralization, providing a promising strategy for developing artificial periosteum biomaterials with favourable bone repair properties [163]. Clay nanoparticles are another popular nanoparticles that have been extensively studied in bone repair. The combination of clay nanoparticles and hydrogel can often enhance mechanical properties and regulate cell adhesion, proliferation and osteogenic differentiation via the release of bioactive ions. Zhai et al. used a hybrid bioink composed of a hydrogen bonding monomer (N-acryloyl glycinamide) (NAGA) and nanoclay to print a 3D composite hydrogel scaffold with excellent mechanical properties and swelling stability. The release of Mg^2+^ and Si^4+^ from the composite scaffold promoted osteogenic differentiation of rat osteoblasts in vitro, and in vivo experiments also demonstrated its ability to facilitate new bone regeneration in rat tibial defects [164]. In another study conducted by Cidonio et al., a synthetic nanoclay (laponite) was blended with GelMA to create a visible light-cured nanocomposite bioink (LPN-GelMA). The addition of laponite increased the shape fidelity and interconnected porosity of the extrusion-bioprinted fibres, which in response promoted the viability and proliferation of human BMSCs cultured within this bioprinted construct. More importantly, the researchers demonstrated its osteogenic capability in the absence of any osteogenic drugs (e.g., DEX) as well as drug (VEGF)-aided angiogenic property in a chicken chorioallantoic membrane (CAM) model, providing a new strategy for bone regeneration [165].

Compared with inorganic nanoparticles, the introduction of polymeric nano-fillers not only gives the hydrogels better biodegradable properties, which has tremendous advantages in regenerative medicine, but also provide a strategy for the loading and release of biomolecules (drugs, growth factors) to achieve enhanced bone repair. Fujioka-Kobayashi et al. developed a cholesteryl group- and acryloyl group-bearing pullulan (CHPOA) nanogel, loaded with BMP-2 and recombinant human fibroblast growth factor 18 (FGF18), and further cross-linked this nanogel with thiol-bearing PEG to form a biodegradable nanocomposite hydrogel. Animal experiments showed that this system improved the efficiency of BMP-2-dependent bone healing in the mouse calvarial defect model via the synergistic effect of the two growth factors, inducing effective bone formation [166]. In addition, nanogels based on smart responsive biomaterials can also exhibit unique swelling or shrinking behaviours in response to external environmental stimuli (e.g., specific temperature, pH), allowing for minimally invasive treatment of bone repair and precise matching of complex bone defects. The most common temperature-sensitive hydrogels are N-isopropylacrylamide (NiPAAM) and its derivatives, which undergo an affinity change with the solvent at a certain temperature (known as the lowest critical solution temperature, LCST), resulting in a state transition, i.e., the sol–gel transition. Since the LCST of NiPAAM is close to the human body temperature (37 °C), smart nanogels developed from it behave as sols at room temperature and become hard solid gels when implanted into the body, thus functioning as a bone tissue engineering scaffold [167].

### Nano-biomaterials for orthopedic implants

For severe fractures or massive bone loss due to high-energy trauma and bone tumour, special endosseous implants (internal fixators, artificial bones, prostheses) are often required to restore normal bone volume and function [168]. The key to determining the implantation status depends on the interaction between the biomaterial and host tissue. Unfortunately, conventional biomaterials have related issues in guiding tissues for implantation at specific sites and influencing cellular functions, e.g., metals have greater stiffness and elastic modulus than physiological bone, leading to stress shielding [169], whereas bioceramics lack ductility and fracture toughness [170]. In addition, polymeric materials suffer from progressive wear and temperature-dependent deformation under loading conditions [171]. Nanomaterials, with their extraordinary physicochemical properties and biomimetic functions, can improve the mechanical properties together with the biocompatibility of the orthopedic implants to maximize the recapitulation of a natural bone-like environment for osseointegration, thereby efficiently reducing fatigue and fracture of the plate as well as wear and loosening of the prosthesis caused by mechanical mismatch at the bone-implant interface [32, 172]. Currently, nanomaterials for orthopedic implants mainly include metals and their alloys, non-metallic materials and composites.

#### Nanoengineered metals

Metallic materials, mainly including Ti-based alloys [173], stainless steel [174], and cobalt (Co)-based alloys [175], are the earliest implant materials developed and applied for internal fixation or arthroplasty in weight-bearing areas of the human body, due to their excellent mechanical properties. Since stainless steel is subject to local corrosion [176], while Co-based alloys release carcinogenic ions in vivo [177], Ti and its alloys occupy an essential position in the engineering of biomedical implants. Ti is light in weight with good fatigue strength and high biocompatibility, making it an ideal medical material for intramedullary nails and artificial joints [178]. The disadvantage of Ti materials is their low hardness and inadequate wear resistance. If wear occurs, it first leads to the destruction of the dense titanium oxide (TiO_2_) film on its surface, followed by the corrosion products of the wear particles into the human tissue. The corrosion products can induce cell death and potential immune response. For instance, the Ti6Al4V alloy contains toxic vanadium (the effects of vanadium compounds have been described as carcinogenic, immunotoxic and neurotoxic, and it has been reported that metal particles less than 10 µm in diameter can be internalized by cells, potentially triggering cytotoxicity, chromosomal damage and oxidative stress) can lead to the failure of the implants [179]. Therefore, in order to improve the osseointegration of Ti alloy, researchers have developed several methods to optimize the implants. One example is inducing more favourable interactions between the metallic implants and native bone through surface modification (Fig. 3D). Specifically, one common approach to improving wear resistance is plasma electrolytic oxidation (PEO) [180]. Studies have shown that coating the Ti alloy surface with PEO coatings can alter its wear pattern to mild abrasive wear, thereby reducing wear volume and wear rate [181, 182]. However, the drawback is that the PEO coating can be easily removed from the surface. To further address this problem, Narayanan et al. modified the Ti6Al4V alloy surface by large pulse electron beam (LPEB) irradiation to reduce the particle size and transform a + b binary into a single a'-martensite phase with a homogeneous microstructure. The LPEB-treated surface had an improved finish and fine organization with uniform tissue distribution, which promoted the discharge characteristics of the PEO process, resulting in higher breakdown potential and final potential, which was conducive to the formation of dense PEO coatings. As a result, the wear resistance of Ti6Al4V alloy treated with LPEB-PEO could be improved by 70.82% [183]. In terms of improving mechanical strength, severe plastic deformation (SPD) effectively refines (submicron or nanoscale) metals by introducing large strain in the deformation process, thus obtaining bulk nanomaterials with high strength and large plasticity at the same time [184]. In contrast, nano-phase Ti created via powder metallurgy (P/M) route possesses higher strength and better ductility than Ti parts processed by SPD techniques, with more particle boundaries and higher surface roughness, thus significantly enhancing the adhesion of osteoblasts [185, 186]. In addition, techniques including electron beam evaporation [187] and anodization [188–191] can generate nano-surface features on metallic implants that improve the morphology and alignment of early osteoblasts to promote osteogenesis in vitro and in vivo. For example, Li et al. created anodized nanotubes with different diameters on Ti6Al4V substrates based on optimized electrolyte hydrodynamic conditions (namely, a uniform velocity and shear stress profile was achieved by anticlockwise adjacent stirring in this study). They then evaluated the osteogenic differentiation capacity of human MSCs on their surfaces by further cell seeding and culture assays. The results showed a significant increase in the expression of osteocalcin and osteopontin as well as mineralization deposition in the 39 nm group [192]. In another example, 3D-printed porous Ti biomaterial covered with nano-tubular surfaces was developed using an optimized anodizing protocol, which was demonstrated to prevent biofilm formation when loaded with middle and high concentrations of Ag ions. Interestingly, even the nanotubes not loaded with Ag ions showed a significant reduction in the number of adherent bacteria on day 1 as well, which might be associated with the bactericidal effect of the high aspect ratio nanotopographies [193]. However, Ryu's study showed that the surface roughness of cobalt-chromium–molybdenum (CoCrMo) alloy accelerated the contact corrosion–fatigue damage on the implant surface [194]. As a result, despite the beneficial effect of surface roughness on the biocompatibility of the implant, it may reduce the lifespan of the implant.

Another modification method is to improve the biocompatibility of the Ti alloy by applying nano-coating on its surface to make it more suitable for the external environment of osteoblasts [195]. Liu et al. prepared lithium (Li)-containing nanoporous coating on the surface of Ti wire porous scaffolds by micro-arc oxidation (MAO), which significantly improved the adhesion and viability of human osteoblasts MG-63 cells and enhanced osteogenic differentiation by activating the Wnt/β-catenin signal pathway. The in vivo experiments also confirmed the sufficient therapeutic effect in the rabbit femoral shaft defect model [196]. In another study, Ren et al. synthesized Ti implantable composites with antibacterial properties, which had a multi-layer structure, with a nano-network composed of sodium titanate on the surface of Ti, and silver nanoparticles between the layered titanate lattice to form a sandwich structure of titanate–AgNPs–titanate. The experimental results showed that this nanostructure could release Ag ions stably and continuously, which contributed to the long-term antibacterial process while maintaining low toxicity to cells, holding broad application prospects in the field of implantable biomaterials [197]. In addition, the tissue regenerative potential of EVs creates the possibility of developing bioactive coatings. Pansani et al. immobilized EVs secreted by decidual mesenchymal stem cells (DEVs) on the implant surface, which not only promoted osteoblast proliferation, migration and deposition of mineral phases, but also directly stimulated the apposition of CaP on the Ti surface, thus improving HA formation and enhancing osseointegration of the implants [198]. Similarly, Chen et al. proposed a coating strategy based on ADSC-derived EVs, and showed that the cell expansion area as well as ALP, collagen I and osteocalcin gene expression were significantly higher in the EV-Ti group than that of pure Ti, suggesting that functionalization of the surface using MSC-EVs could enhance the osteoinductive activity of the implants [199].

Nonetheless, most metallic materials do not degrade and are prone to stress shielding, modulus mismatch and immune reactions in surrounding tissues [32, 200, 201]. As a candidate metallic material, magnesium (Mg) alloys have shown promise in developing novel orthopedic implants due to their favourable biodegradable features and suitable mechanical properties [202]. It has been demonstrated that Mg can accelerate the growth of bone tissue [203]. However, one of the major concerns of this alloy is the rapid and inhomogeneous corrosive degradation, and the high rate of Mg ions released from implants in the human body may be toxic [204]. To address this issue, many coating systems have been introduced to improve the corrosion resistance of Mg biomaterials [205–207]. For instance, Tian et al. successfully deposited nHA coatings on the surface of Mg substrates using a transonic particle acceleration (TPA) process, and performed in vitro degradation and mechanical properties studies in revised SBF for 6 weeks. The results showed that the coating effectively reduced the degradation rate of Mg-based implants and maintained 86–90% of compressive strength (much higher than the 66% strength maintained by uncoated Mg), which still met the mechanical requirements of load-bearing implants [208]. However, there is also evidence that the effectiveness of the coating may be impacted by the inconsistence in Mg surface topography, i.e. the biomimetic coating technique can prevent the corrosion occurring within smooth polished samples, but fail in Mg substrates having rough surface, such as porous implants prepared via casting [209]. This may be due to the inconsistency of the coating layer produced by the rough surface samples during the actual coating process resulting in cracks and defects, which can lead to corrosion underneath the coating and consequently to reduced coating adhesion. In response, Waterman and his team improved the properties of these coatings using an electrochemical assisted deposition (ECAD) coating of calcium hydroxide (Ca (OH)_2_), which deposited additional protective CaP at the corrosion site through the dissolution of Ca (OH)_2_ in reaction with phosphate ions in the body fluid [210].

#### Nanostructured non-metallic materials

##### Nano-ceramics

Given that the chemical composition of certain bioceramics is similar to the minerals of native bone, and their reaction with physiological fluids produces a strong bond to soft & hard tissues, thus increasing the osseointegration between implants and bone, i.e. good biocompatibility with bone tissue, bioceramics have been widely used in orthopedics [211]. Based on the reaction degree between implant material and biological tissue, bioceramics can be categorised into bioactive ceramics (HA, TCP, bioactive glass (BG)) and bioinert ceramics (mainly composed of metal oxides, of which aluminium, zirconium and Ti are the most widely used) [211]. However, bioactive ceramics suffer from low strength and brittleness and cannot be used for repair of high-loading bone defects, while bioinert ceramics have low fracture toughness and form a fibrous tissue interface with bone in vivo, which also limits the osseointegration [170]. To solve these problems, researchers have obtained nano-ceramics by further reducing the grain scale and pore size based on micron-size bioceramics [211]. In addition to exhibiting material properties similar to those of nanoengineered metals (e.g., increased surface roughness can promote osteoblast adhesion) [212–214], nano-ceramics also have some unique surface properties, such as special surface topography, improved surface wettability and increased surface grain boundary number, which play a great role in ameliorating osseointegration [215–217]. As a result, nanoceramics are extensively applied in bone filling and bonding, as well as replacement materials to improve the antimicrobial ability, wear resistance and mechanical properties of implants [170, 218].

**Fig. 5:**
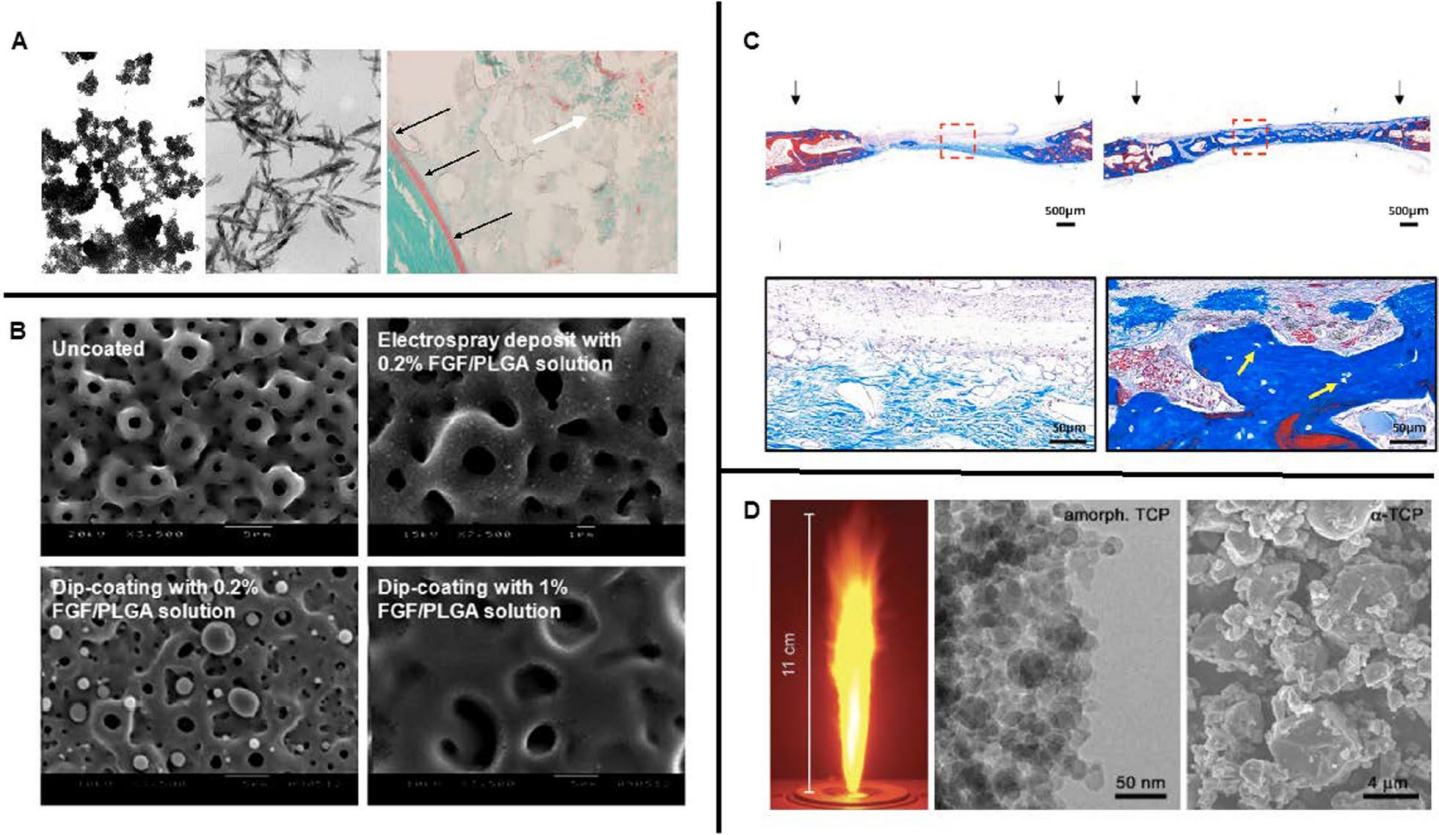
**A** Histological analysis showed well-structured cancellous bone tissue [220]. **B** SEM images of Ti discs coated by ESP technique [254]. **C** MT histological staining revealed that the area covered by new bone in the Ta–PLA group was almost twice as large as the covered area in the bare PLA group [223]. **D** TEM images of amorphous TCP nanoparticles fabricated by spray flame [232]

Taking HA, the most versatile biomaterial in bioactive ceramics, as an example, as the main inorganic component of human hard tissues (bones and teeth), it has good biocompatibility. However, the inadequate mechanical properties and low fatigue resistance limit its application in high-pressure situations [219]. By mimicking the mineralization process of physiological apatite, researchers generate HA precipitates from a mixture of carbonates and phosphates via double decomposition reaction under conditions similar to the physiological conditions of the human body, harvesting nanophase (materials with grain sizes less than 100 nm) HA through further high-temperature calcination. The development of nHA overcomes the disadvantages of conventional HA, such as low strength and slow degradation. nHA also has enhanced surface properties, which are conducive to cell adhesion and growth, due to its closer structure to that of crystalline HA in native bone. For example, Huber et al. injected a nanocrystalline HA paste (Ostim) into the cancellous bone of fracture patients for histological evaluation, and the results showed that Ostim was extensively absorbed with good tissue integration (Fig. 5A) [220]. Another experiment demonstrated that the addition of Ostim to TCP/HA ceramic granules could reduce the penetration of bone cement while maintaining the original stability of the acetabular cup, without compromising the biocompatibility of the material [221]. Besides, Iskandar et al. obtained nHA-coated Mg-based implants by a transonic particle acceleration (Spire Biomedical) process, and further confirmed that this coating could reduce the degradation of Mg while improving its integration with bone tissue under standard cell culture conditions [222].

In addition, to achieve better histological response and satisfaction, nHA complexes can be obtained via the addition of a second phase or multiphase material. The composites take full advantage of the biocompatibility of nHA and the superior processing properties of nanofillers, better mimicking the structure of native bone.

##### Nano-polymers

Nano-polymers offer some unparalleled advantages over the implant materials mentioned above, such as their customizable mechanical properties and degradation rates, as well as the ability to be made into injectable materials that harden in situ. In addition, their physical properties are very similar to those of proteins found in soft & hard tissues, which can be modified functionally through biochemical reactions, providing a wide range of properties to interact with different types of cells [211].

PLA is a class of highly hydrophilic polymeric degradable material, which can be fabricated into rods, screws and plates for fixing bones with relatively rapid fracture healing, as well as for arthroscopic reconstruction of the ruptured anterior cruciate ligament of the knee. It is also a promising scaffold material for cell culture in the field of bone tissue engineering. As an internal fixation material, the use of PLA is less likely to abrade soft tissues during surgical operations. Its mechanical strength can be properly attenuated with the healing of bone tissue, thus allowing normal stress stimulation of the fracture end without the disadvantages of stress masking that exist with metallic materials. However, the insufficient osteoconductivity of PLA was a significant challenge, which was addressed by coating highly biocompatible tantalum (Ta) on the surface of electrospun PLA NFs using a direct current (DC) sputtering method in Hwang et al.’s study. In vitro cell tests showed that Ta-PLA membranes promoted the attachment, proliferation and differentiation of preosteoblasts compared to bare PLA (Fig. 5C) [223].

Poly (methyl methacrylate) (PMMA) is the basis of bone cement, which consists of radial particles of 1 ~ 3 μm in size. The incomplete dispersion of these particles forms agglomerates of 50–200 μm diameter, where large voids reduce the fracture toughness of PMMA. Studies have shown that replacing micrometre size radiopacifying particles with aluminium oxide particles resulted in a significant reduction in the volume of bone cement and a significant increase in tension properties, making PMMA nano-cements a promising alternative to conventional bone cement in orthopedic practice [224].

#### Nanocomposites

Since it is difficult for a single type of material to adequately meet the specific needs of bone repair in complex environments, researchers have synthesized organic/inorganic, ceramic/polymer composites by combining several materials appropriately, taking into account the advantages and disadvantages of each component, in order to achieve optimal outcomes in practical applications.

##### Ceramic/Polymer composite bone biomimetic material

Studies have shown that synthetic composites that approximate the size and morphology of inorganic particles and organic phases of bone can achieve better osteoconductivity, evidenced by the improved osteoblast function in PLA/calcium metaphosphate composite developed by Jung et al., compared to PLA scaffolds alone [225]. Besides, incorporating biodegradable polymers compensates for the slow degradation rate of ceramic materials, making them more suitable than single components as orthopedic implant materials. For instance, taking advantage of the function of biological systems to store and process information at the molecular level, nHA/collagen matrix nano-biomimetic materials can be prepared for bone defect filling by simulating native bone biomineralization and self-assembly mechanisms in vitro. Since nHA crystals are uniformly deposited on collagen, which can be easily recognized and utilized by the human body, and collagen has the biological property of inducing tissue cell growth, accelerating fracture healing and callus formation. As a result, this nano-biomimetic material can be used for repairing bone defects, or combined with seed cells and growth factors as bone tissue engineering matrix material to improve the quality of repair, especially for segmental bone defects [226].

##### Nanostructured calcium phosphate cement (CPC)

CPC is a self-setting synthetic bone implant material composite of CaP, which can be surgically implanted into the human body or injected directly into the cavity of bone defects (the paste can self-harden in-situ to form HA with similar structure and composition to human hard tissues), with the characteristics of arbitrary shaping and good biocompatibility. Due to its pH-neutral composition and absence of polymers or plasticizers, it can be used in long-term contact with surrounding tissue without toxic reactions [227]. However, the performance of CPC in some aspects (e.g., compressive strength, curing time, degradation rate) is still difficult to meet the clinical requirements. Thus it is mainly used to fill or repair bone defects, and replace certain non-load-bearing bones [228, 229]. Interestingly, it has been shown that nanostructured CaP biomaterials exhibit better physicochemical and biological properties than those of conventional size, which are more similar to bone nanocrystals [230, 231]. Therefore, the study of CPC modification has become an important direction in bone repair materials. One approach to fabricate nano-CPC is to limit its beginning particle size at the nanoscale level. For example, Brunner et al. used a flame spray synthesis method to fabricate amorphous TCP nanoparticles (Fig. 5D). Due to the higher specific surface area, amorphous-TCP significantly accelerated the conversion to calcium-deficient hydroxyapatite (CDHA) during the self-hardening process of CPC [232]. In another study, Ginebra et al. also demonstrated that the reduction in particle size could lead to a significant decrease in setting time and accelerate the hardening of the cement without substantially affecting the final strength of the cement [233]. The alternative modification design is to add other components into CPC, forming synthesized biocomposites with optimized properties. Roozbahani et al. developed a nano-CPC with less setting time and higher mechanical properties using silica-stabilized α-TCP (98 wt.%) and HA (2 wt.%) as the powder phase and NaH_2_PO_4_ solution (2.5 wt.%) as the liquid phase of the cement, which exhibited significant enhancement in proliferation and adhesion of MG63 cells [234]. Similarly, Xu et al. combined nano-silica-fused whiskers with CPC and added these composite particles as fillers to the resin matrix, resulting in a great improvement in strength, with flexural strength up to 164 MPa, which was three times stronger than the control group containing only CPC fillers, and was close to the mechanical properties of cortical bone. Moreover, osteoblast-like cells (MC3T3-E1) responded well when adhering to the nano-apatite structure of CPC [235]. In addition, Shu's team fabricated a nano-doped CPC delivery system (nano-γ-PGA/β-TCP/CaP ceramic) as a carrier material for controlled-release of dual growth factors (IGF-1 and BMP-2), which could not only resist washout, but also could affect the release kinetics of the drugs by adjusting the composition ratio of the composite, thus promoting bone healing and bone ingrowth in low-dose therapy, with promising applications in the treatment of infected bone defects [236].

##### Ceramic/carbon nanostructures

Carbon nanostructures (e.g., carbon NFs, CNTs, graphene, NDs) have been widely studied and applied as reinforcing materials for orthopedic implants in recent years due to their generally ultra-high mechanical strength. For example, the physical properties of artificial bone were greatly enhanced by the addition of carbon NFs, which also improved the adhesion of osteoblasts, leading to an accelerated fusion between bone and the implant surface, with an improved cellular proliferation of osteoblasts as well as the synthesis of ALP, accumulating more ECM [237]. In another example, CNTs had been reported to improve the stiffness, tensile strength and impact toughness of the polypropylene (PP)/nHA nanocomposites with good biocompatibility to human bone [238]. In addition, Baradaran et al. prepared reduced graphene oxide (rGO) reinforced composites by hydrothermal method and demonstrated that the elastic modulus and fracture toughness of the sintered samples improved with increasing rGO content compared to pure nHA [239].

##### Nano-coatings

Nano-coating modification of metallic substrates is an important current research direction in orthopedic implant materials. For example, researchers have successfully constructed a physiological transition layer that mimics the native bone tissue composition at the interface between the implants’ surface and surrounding host bone to enhance osteoblast function and promote bone regeneration, thereby improving the osseointegration [240]. Nano-coatings can effectively modify the dominant surface properties (e.g., ion release, tribological characteristics, corrosion resistance) without affecting the properties of the bulk material [241], ensuring the proper longevity and stable fixation of the implants. For instance, the deposition of glycidyl methacrylate (GMA) nanolayer on Ti surface showed increased protein adsorption, higher ALP activities, and better calcium deposition [242], while coating nanostructured oxide on stainless steel and Ti alloy substrates significantly improved their corrosion resistance [243]. In addition, CaP has the optimal biocompatibility as the composition of native bone tissue, which makes it a popular coating for Ti-based implant materials [244, 245]. Nevertheless, the traditional coating technology has corresponding technical defects, such as plasma spraying cannot control the thickness of the coating as well as the surface morphology, and the elevated temperature during the spraying process would alter the structural properties of the coating, resulting in coating peeling or even dissolution and ultimately leading to the reduction of the bonding strength between the coating material and the substrate [246]. In contrast, as an emerging surface modification technology, microwave technique (MWT) not only eliminates the high operating costs of laser-based coating [247], but also has a higher material deposition rate than chemical vapour deposition (CVD) and physical vapour deposition (PVD) [248]. Meanwhile, instead of the conduction heating of thermal spray processes, the heat generation pattern of MVT uses internal heating by coupling the coating powder with microwaves, which reduces the thermal gradient and minimizes the heat-affected area, avoiding exposing the substrate and coating to rapid oxidation, metallurgical transformation and adverse residual stresses, providing the possibility to meet additional tailored properties [249]. Some scholars applied multiple preparation methods simultaneously under suitable conditions so that their advantages complemented each other, such as the co-application of MAO and electrophoretic deposition (EPD), which formed a ceramic film dominated by substrate metal oxides on the surface of Ti, relying on the transient high temperature and high-pressure effect generated by arc light discharge. The benefit was the strong bonding with the metallic substrate, which precisely compensated for the deficiency of the EPD method [250].

In order to synthesize coatings of implant materials with more desirable biological properties, an increasing number of researchers have turned their attention to the study of composite coatings. Prodana et al. developed a composite ceramic coating on Ti plates using TiO_2_ nanotubes obtained by anodizing MWCNTs functionalized with -COOH groups and HA, which demonstrated an enhanced osteoblast response in terms of adhesion, viability and proliferation [251]. In another study, Ahmed et al. fabricated calcium carbonate (CaCO_3_)/MWCNTs/CS nanocomposite coatings for surface modification of Ti6Al4V alloy via electroless deposition and reported improvement in bioactivity as well as corrosion resistance [252]. In the study conducted by Sarao et al., proprietary thermal spray equipment was used to deposit HA powder with TiO_2_ composite coating on Ti alloy. Electrochemical studies showed a significant improvement in the corrosion resistance of the substrate after coating deposition [253]. Shim et al. deposited fibroblast growth factor-2 (FGF-2) loaded poly (lactide-co-glycolide) nanoparticles on anodized Ti discs by an electrospray deposition technique (Fig. 5B), and demonstrated in vitro and in vivo that the nanoparticle composite coating allowed continuous delivery of growth factors as well as significantly increased the osseointegration value of the implants to promote bone regeneration [254]. In addition, GO/CS [255] as well as NDs-reinforced alginate–bioactive glass films [256] had been fabricated on the surface of Ti implants by EPD technique, and in vitro viability assay by MG-63 showed that both of these nanocomposite coatings were highly biocompatible.

## Nanomedicine in cartilage repair

Articular cartilage is a dense connective tissue covering the joint surface composed of one single cell type (chondrocytes) with its ECM, which plays an essential role in cushioning and minimizing friction during joint movements [257]. Due to the lack of blood supply and local progenitor cells, cartilage has a minimal self-repair capacity and is prone to injury by external forces or aging, resulting in developing joint diseases; the most typical and common one is OA. With the gradual aging and increased obesity in our society, the risk of cartilage damage in the population has increased significantly. The existing treatments are inadequate to cope with this burden [258]. Thus, tissue engineering (aiming to fabricate biological substitutes to replace damaged tissue and recapitulate the development or repair processes of native tissue) has been studied intensively in cartilage repair.

The term “repair” in cartilage tissue engineering encompasses two separate concepts, one of which is a replacement, such as mosaicplasty (i.e., osteochondral transplantation), a common resurfacing technique that restores the partial function of osteochondral units by removing cylindrical plugs of healthy cartilage with subchondral bone and implanting them into the defective area like a mosaic pattern [259]. This method is appropriate for small lesions, aiming to relieve pain and prevent the disease progression [260]. The other one is regeneration, which refers to the complete restoration of damaged tissues to their normal form. It is usually used for extensive symptomatic defects enclosed by non-osteoarthritic cartilage [261], such as autologous chondrocyte transplantation (ACI) that induces chondrogenesis by injecting autologous chondrocytes under a periosteal patch (the first generation) or replacing the periosteum with a collagen membrane (the second generation) [262]. Clinical trials have reported favourable histological results, as well as potential complications, including the loss of transplanted cells after surgery and the heterotopic ossification of periosteum [263, 264]. In response, researchers developed the third generation of ACI, namely matrix-induced autologous chondrocyte implantation (MACI), which overcame the shortcomings of the first two generations by pre-implanting cells in a 3D matrix (biomaterial scaffold) before transplanting them into the lesion sites [265]. However, early scaffolds could only simulate cartilage in a simple way without reproducing its natural stratified structure and function due to material limitations. As a result, the quality of regenerated tissue is inferior to hyaline cartilage in terms of morphology or histochemistry, failing to meet the requirements of durable compression resistance and high loading [266]. In recent years, with the rapid development of material science, ECM substitutions using nanocomposite hydrogels and nano-structured scaffolds have been introduced with enhanced therapeutic effects (Table 2).

**Table 2:** Summary on properties and applications of nanocomposites for cartilage tissue repair

Material	Properties	Application	Significance	Refs.
*PCEC/alginate*	Injectable hybrid scaffold using biodegradable porous microsphere as the cross-linker carrier	Repair full-thickness cartilage defects in a rabbit model	This injectable scaffold may be useful to meet different shape defects and regrow cartilage layers by a minimally invasive approach	[286]
*CMs/CMC-OCS*	Injectable CMC-OCS hydrogel containing CMs developed via the Schiff’ base cross-linking reaction	Encapsulate bovine articular chondrocytes in vitro	CMs-embedded CMC-OCS hydrogels have potential as injectable drug and cell delivery systems in cartilage tissue engineering	[287]
*TGM/PAMAM/Fe*_*3*_*O*_4_	Injectable nanocomposite hydrogels containing pNiPAAm-based TGM, PAMAM-based macromers, and Fe_3_O_4_ nanoparticles	Encapsulate WRN cells	The integration of the nanoparticles made the hydrogel responsive to a magnetic field, indicating the feasibility of utilizing an external device to deliver spatiotemporally-controlled mechanical stimuli to encapsulated cells	[281]
*gelatin/PLA*	Porous 3D scaffold containing electrospun gelatin/PLA nanofibers	Repair the cartilage defect in rabbits	The composite scaffold possessed porous and nanofibrous structure, which could mimic the structure of native ECM, improving the growth of chondrocytes in vitro	[291]
*gelatin/PLLA*	Embedding gelatin onto the surface of nano-fibrous PLLA scaffolds developed by TIPS using an electrostatic layer-by-layer self-assembly technique	Culture MC3T3-E1 osteoprogenitor cells	Developing a novel procedure for surface modification of nano-fibrous PLLA scaffolds that were advantageous for cell adhesion and proliferation	[344]
*PLCL/nHA*	Composite scaffolds fabricated by TIPS followed by a freeze-drying technique	Study the in-vitro degradation of nanocomposites for use as scaffolds in bone engineering	The introduction of nHA could modulate the degradation rate of PLCL scaffolds	[293]
*NaOH-treated PLGA*	Underlying material properties obtained via chemical etching techniques using NaOH include a more hydrophilic surface, increased porosity, and a greater degree of nano-roughness	Culture human articular chondrocytes in vitro	Demonstrating the potential use of NaOH-treated PLGA for enhanced articular cartilage repair	[295]
*PCL-b-PLLA*	Nanofibrous scaffold created via combining TIPS with salt-leaching methods	Culture chondrocytes in vitro	Compared with solid-walled scaffolds, nano-fibrous scaffolds have larger specific surface area and protein adsorption, on which the chondrocytes are cultured in a spherical shape with enhanced viability and proliferation, making them potentially excellent scaffold materials for cartilage tissue engineering	[296]
*PLLA/SF*	Nanofibrous scaffold fabricated by electrospinning	Culture rabbit articular chondrocytes in vitro	The PLLA/ SF scaffold is more conducive to in vitro formation of cartilage-like new tissues than the unmodified PLLA scaffold	[297]
*PLLA/gelatin/GAG*	GAG-containing composite nanofibers consist of co-electrospun PLLA/gelatin	Culture BMSCs and chondrocytes	The PLLA/gelatin/GAG blended nanofibers displayed significant increases in hydrophilicity, cell proliferation and chondrogenic differentiation	[298]
*gelatin-PCL/DCECM*	Composite scaffolds containing electrospun nanofibers and DCECM	Repair cartilage defects in New Zealand white rabbits	This composite scaffold has stronger structural stability and higher chondrocyte proliferation rate, which is a promising tissue engineering scaffold for cartilage regeneration and cartilage defect repair	[299]
*PAA-Alg-Si*	Composite hydrogels, combined with nano-silica	Culture ADSCs	Hydrogels incorporated with silica show a significant increase in compressive strength and fracture toughness, while having considerable hydrophilicity, which is in accordance with the nature of soft tissues such as cartilage	[300]
*chitosan/alginate*	Composite scaffold consist of alginate solution (containing BMP-7) and chitosan nanoparticles (containing TGF-β_2_)	Culture MSCs	The dual growth factors (BMP-7/TGF-β_2_)-loaded nanoparticle/hydrogel system showed a controlled release of both growth factors, providing desirable growth factor delivery kinetics for cartilage regeneration, as well as the chondrogenesis of MSCs	[304]
*GO/PDLLA*	Photopolymerizable PDLLA hybrid hydrogel incorporated with GO	Culture hBMSCs	With the presence of GO, the hydrogel scaffold supported in vitro TGF-β_3_ retention for up to 4 weeks and enhanced scaffold compressive stiffness, on which hBMSCs were encapsulated with higher chondrogenic gene expression and cartilage ECM production	[305]

PCEC, amphiphilic poly (ε-caprolactone) − b-poly (ethylene glycol) − b-poly (ε-caprolactone); CMs, chitosan-based microspheres; CMC, carboxymethyl chitosan; OCS, oxidized chondroitin sulfate; TGM, thermogelling macromers; PAMAM, polyamidoamine; pNiPAAm, poly (N-isopropylacrylamide); WRN, Wnt Rspondin Noggin; PLA, poly (lactic acid); ECM, extracellular matrix; PLLA, poly (L-lactic acid); TIPS, thermally induced phase separation; PLCL, poly (lactide-co-E-caprolactone); nHA, nanohydroxyapatite; PLGA, poly (lactic-co-glycolic acid); PCL, poly (ε-caprolactone); SF, silk fibroin; GAG, glycosaminoglycan; BMSCs, bone marrow mesenchymal stem cells; DCECM, decellularized cartilage extracellular matrix; PAA, poly (acrylic) acid; Alg, alginate; BMP-7, bone morphogenic protein 7; TGF-β_2_, transforming growth factor-beta 2; GO, graphene oxide nanosheets; PDLLA, photopolymerizable poly-D, l-lactic acid/polyethylene glycol

### Nanocomposite hydrogels

Among numerous biomaterials, hydrogel is considered an ideal material for cartilage repair due to its similar properties to ECM [267]. When injected into the injury site of cartilage, hydrogel precursor solution containing cells or growth factors can perfectly fill the defect and rapidly polymerize to form a solid gel within a short period of time, thus achieving in situ cartilage repair [268]. Compared with engineered cartilage tissue scaffolds fabricated through the traditional process, the injection approach is minimally invasive, with good dispersion of bioactive factors and the usability to match irregular defects [269]. Common hydrogel materials include polysaccharide biomaterials such as agarose [270], chitosan [271], alginate [272], hyaluronic acid [273], silk [274], and protein biomaterials including collagen [275] and fibrin [276]. In addition, synthetic materials have also been widely studied in this field due to their excellent mechanical properties, improved processing potential and reduced batch to batch variation, such as PEG [277], Poly (vinyl alcohol) (PVA) [278], NiPAAm [279]. However, single-component hydrogel materials are often deficient in performance [280]. Therefore, an increasing number of studies has applied the combination of nanoparticles with biomaterials to form nanocomposite hydrogels to improve their mechanical strength, stability and functionality. Besides, the embedding of nanoparticles can effectively improve the hydrogel network's structural properties and pore size, promoting cell attachment, which stimulates cell growth and guides tissue regeneration.

*Metal oxide NPs:* Adedoyin et al. obtained an injectable nanocomposite hydrogel using inorganic iron oxide (Fe_3_O_4_) nanoparticles covalently combined with pNiPAAm and PAMAMs, which not only reduced the gelation time, but also responded to external magnetic fields to stimulate cellular activity and in situ regeneration [281]. In another study, Huang et al. prepared a magnetic nanocomposite hydrogel with gelatin, β-cyclodextrin and Fe_3_O_4_, which exhibited excellent superparamagnetic properties, and sufficient mechanical strength with high biocompatibility. In vitro co-culture of this hydrogel with BMSCs under pulsed electromagnetic fields promoted the differentiation of BMSCs into chondrocytes, and in vivo experiments with rabbits also showed improved cartilage repair effects [282].

*Carbon-based NPs:* The addition of carbon-based nanoparticles can enhance the mechanical properties, lubrication and biocompatibility of nanocomposite hydrogels. Specifically, Cao et al. successfully fabricated GO homogeneously covered with HA particles via the dual-anchoring effect of PEG, and further encapsulated them into PVA hydrogels. The experimental results showed that HA with sharp edges of GO coating significantly improved the compressive deformation resistance and the lubrication property of the composite. More importantly, improved cell (mouse BMSCs) proliferation and cytocompatibility were observed, demonstrating the potential in cartilage replacement [283].

*Inorganic NPs:* Inorganic clays are often used to prepare nanocomposite hydrogels with enhanced mechanical properties due to their layered structure and large specific surface area, facilitating the entry of polymer molecules into the nanoparticle lamellae to form a hybrid system with an intercalation structure. For example, Bonifacio et al. used mesoporous silica and sodium-calcium bentonite to modify the gellan gum-based hydrogels, which significantly improved their mechanical properties and showed excellent performance cytocompatibility as well as antibacterial properties in vitro and in vivo [284]. In addition, due to the advantages of high modulus, large specific surface area, and versatile functionalization, silica nanoparticles as crosslinker to bind with hydrogels is also considered as a superior method for toughening hydrogels with great potential in cartilage tissue repair [285].

**Fig. 6:**
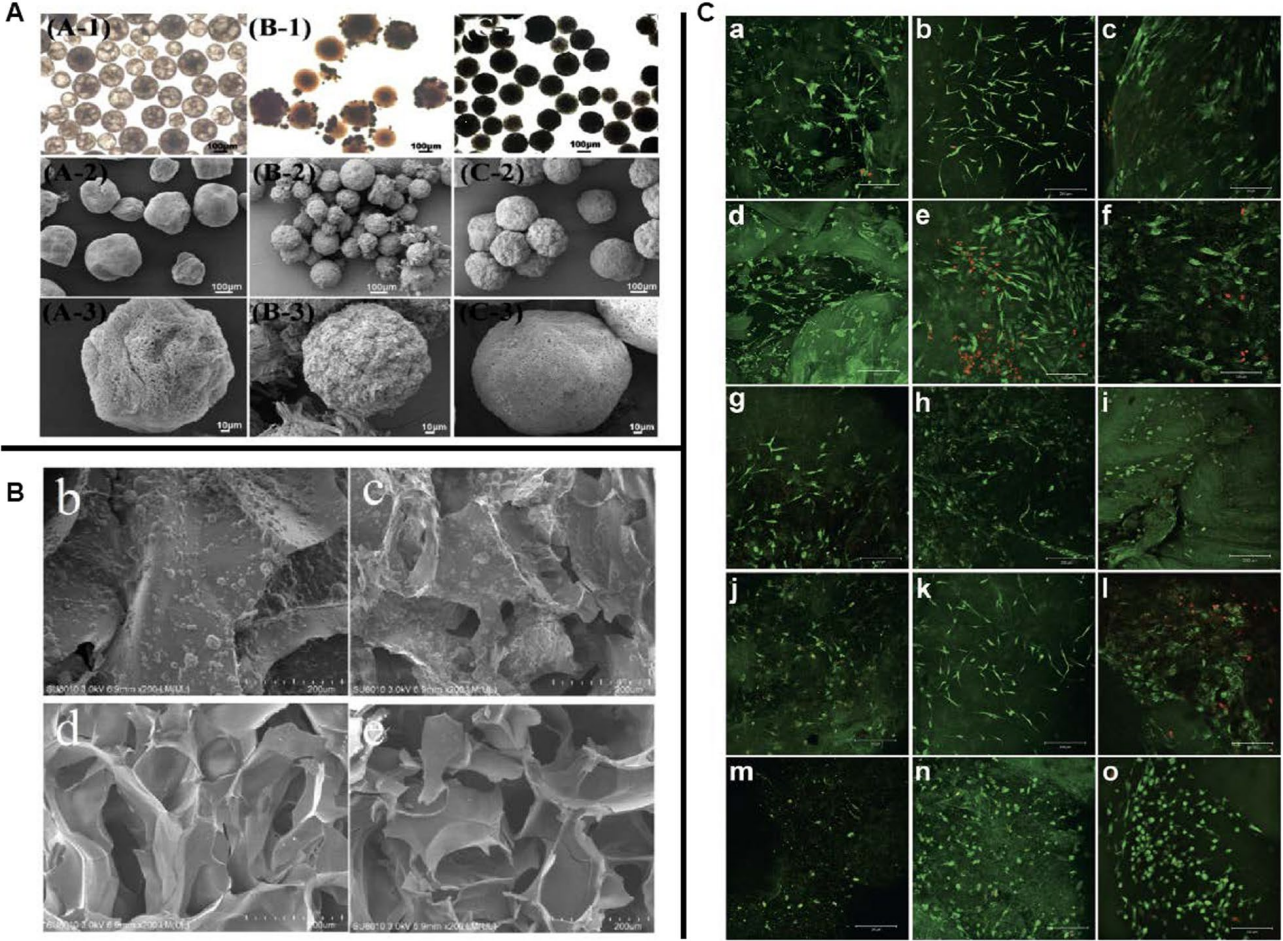
**A** SEM images of porous PCEC microspheres [286]. **B** Porous structure of CMs/gel after 7/14 days of incubation (SEM images) [287]. **C** Confocal microscopic observations indicated that the addition of PCL particles to the scaffold improved cell differentiation into the chondrogenic lineage, resulting in a lower proliferation rate [288]

*Polymeric NPs:* Liao et al. developed a novel injectable 3D alginate hydrogel using PCL − PEG − PCL (PCEC) microspheres as carriers for calcium gluconate (Fig. 6A). With the release of calcium gluconate, chondrocyte/alginate suspensions and porous microspheres were converted into gels, effectively mimicking the architecture of ECM. In vitro and in vivo results indicated that the composite hydrogel had excellent properties, including pore connectivity, high compressive modulus, good formability and favourable degradability, which could be a suitable matrix for cartilage tissue engineering [286].In another study, Fan et al. prepared chitosan-based microspheres (CMs) loaded with bovine serum albumin (BSA) using emulsion cross-linking, and embedded them into hydrogels of water-soluble carboxymethyl chitosan (CMC) and oxidized chondroitin sulfate (OCS) to generate a composite CMs/gel scaffold. Experimental results demonstrated that the embedded CMs could enhance the mechanical properties and bioactivity of gel scaffolds, which exhibited a lower swelling rate and slower degradation than control hydrogel without the addition of CMs (Fig. 6B) [287]. Moreover, In addition, it had been shown that incorporating PCL particles into collagen/ hyaluronic acid/fibrin composite hydrogels helped to slow down the degradation of the gel to achieve adequate systemic stability and promote the differentiation of MSCs into cartilage lineages (Fig. 6C) [288].

#### Nanostructured scaffolds

**Fig. 7:**
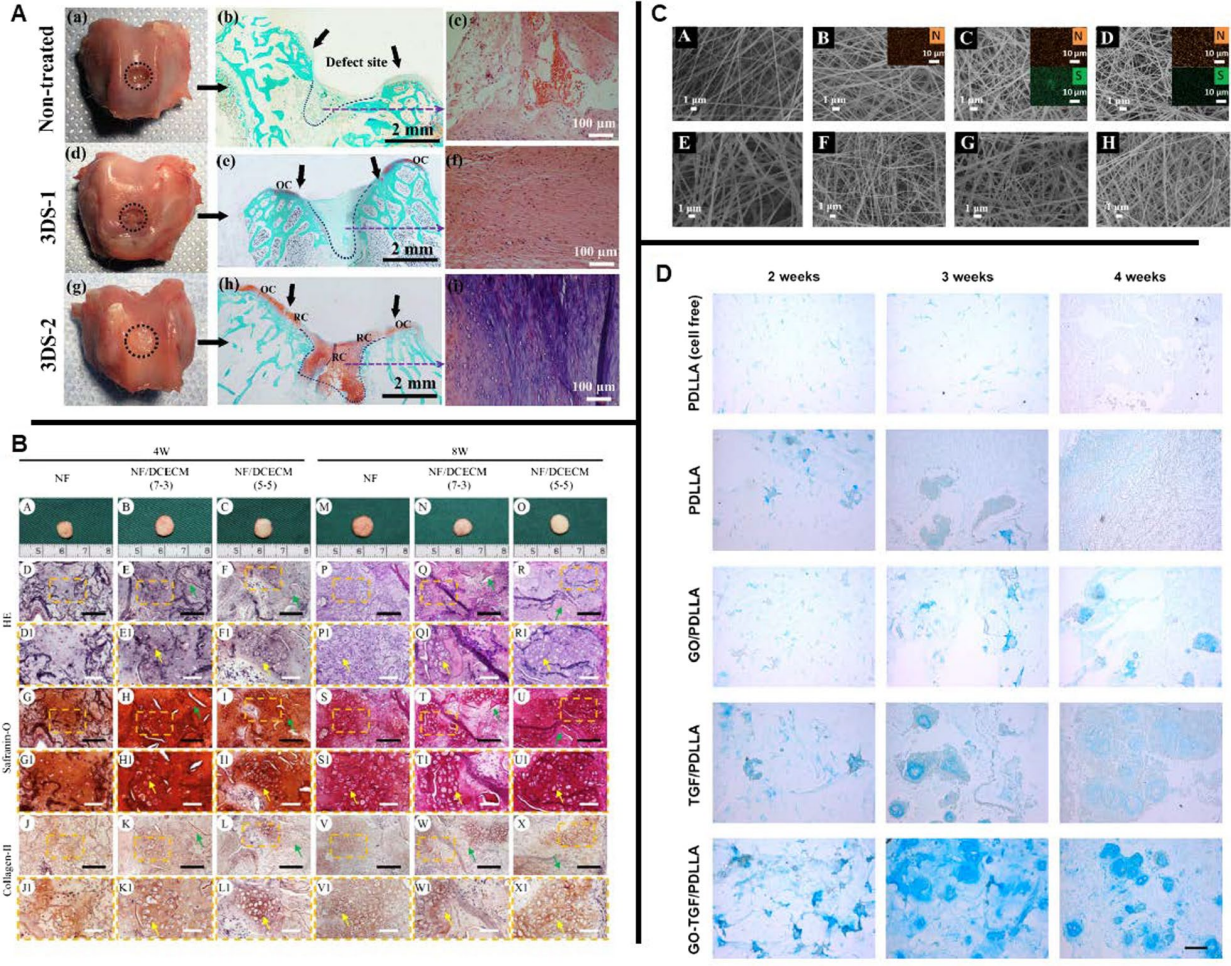
**A** Rabbit articular cartilage macroscopic images and histological analysis at 12 weeks after surgery: the defect in 3DS-2 group was filled with uniform cartilage-like tissue [291]. **B** Histologically, the NF/DCECM scaffold showed typical cartilaginous lacuna features [299]. **C** SEM observations on fibre morphology of various electrospun nanofibers [298]. **D** Histological analysis indicated increased cartilage matrix deposition in the hBMSC laden GO-TGF/PDLLA hydrogel [305]

Nanostructured scaffolds are 3D engineered tissues (NFs or nanoporous polymer matrices) manufactured by electrospinning, self-assembly, and phase separation processes, which are implanted into cartilage defects to simulate the highly porous and pore-connected microenvironment of the human tissue, providing suitable mechanical support and physicochemical stimulation for the binding of seed cells along with growth factors. However, normally, it is difficult for a single material to mimic the components of natural ECM. Hence, most of the relevant research is on the composite application of several materials to meet the optimal conditions for cartilage regeneration, including:

*Favourable biocompatibility:* Synthetic polymeric materials are widely used in cartilage tissue engineering scaffolds due to their superior controllability over physicochemical properties. That being said, the surface of synthetic polymers lacks certain receptor-specific binding bioactive molecules of natural materials, such as RGD sequences that are important for cell adhesion and growth migration, and functional differentiation [289]. Therefore, scaffolds derived from synthetic materials such as PLA, PGA, PLGA are mostly modified with natural polymers or functional groups to create a simulated natural biochemical environment suitable for cell survival. For example, Hsu et al. demonstrated that additional recombinant proteins containing RGD together with nano-sized CDHA to alginate gels facilitated the chondrogenesis of rat adipose-derived MSCs and human placenta-derived MSCs in PLGA scaffolds [290]. Chen et al. reported an electrospun gelatin-PLA nanofibrous 3D scaffold, which could mimic the natural ECM structure and exhibit superabsorbent property and excellent cytocompatibility, to promote the growth of chondrocytes in vitro (Fig. 7A) [291].

*Controllable degradation rate:* The ideal degradability requires an excellent initial morphology of the scaffold after implantation, which is conductive to cell ingrowth, and rapid degradation of the material after 4 weeks to prevent stress shielding. During this process, the scaffold will be gradually replaced by the newly generated ECM until it is finally completely degraded and reabsorbed, maintaining the overall structural integrity [292]. Material compounding is the standard approach to control the degradation rate of the scaffold. For example, it has been shown that nHA could modulate the degradation rate of poly (lactide-co-E-caprolactone) (PLCL) scaffolds due to its ability to absorb water molecules that penetrate into the solid phase. Furthermore, nHA particles can uptake degradation products in the form of monomers and oligomers, further decreasing the autocatalytic degradation of the polymer phase. In parallel, the addition of nHA can reinforce the mechanical properties of the nano-complex as well, making them osteoconductive [293].

*Suitable pore size and porosity:* Studies have shown that proper pore size and porosity allow for cell migration and intercellular signalling, thus contributing to the maintenance of chondrocyte function [294]. For instance, Park et al. obtained chemically etching 3D nano-scaffolds with improved porosity by NaOH treatment of PLGA scaffolds and demonstrated their enhanced effects on chondrocyte adhesion and proliferation [295]. In another study, He et al. created PCL-block-PLLA nanofibrous scaffolds with high porosity for culturing chondrocytes in vitro using TIPS techniques. Their result confirmed that these scaffolds were superior to solid-walled scaffolds in terms of protein adsorption, collagen II and aggrecan mRNA expression levels, resulting in the increased secretion of cartilage-specific ECM [296]. Moreover, Li et al. prepared PLLA/silk fibroin (SF) nanofibrous scaffold by electrospinning and found that when the ratio of PLLA to SF was 60:40, this scaffold had a smaller nanofiber diameter, which was closer to the size of biomolecules in natural cartilage ECM [297]. Furthermore, Zhao et al. fabricated a synthetic/natural hybrid polymer fibrous network composed of PLLA, gelatin and GAG by electrospinning (Fig. 7C). This nanofibrous network had a uniform morphology, average diameter < 300 nm, high porosity and surface roughness similar to that of native bone, which better simulated the ECM structure, effectively promoting cartilage repair [298].

*Appropriate mechanical properties:* Since articular cartilage is under constant loading, the mechanical mismatch between the implanted scaffolds and surrounding tissues may impede cartilage regeneration at the defective area. Therefore, in addition to pursuing the highest possible pore size and porosity, giving the scaffolds mechanical properties similar to those of native cartilage is another important task in cartilage tissue engineering. Li et al. fabricated a composite 3D scaffold with biomimetic structure and bioactivity using electrospun gelatin-PCL NFs and decellularized cartilage extracellular matrix (DCECM). The scaffold had an improved mechanical properties and structural stability as compared to DCECM scaffolds with enhanced chondrocyte proliferation rate. More importantly, in vitro and in vivo results showed the enhanced cartilage regeneration (Fig. 7B) [299]. In another study, the incorporation of nano-silica into the poly (acrylic) acid/alginate/silica (PAA-Alg-Si) hydrogel scaffold prepared by Lin et al. greatly increased the compressive strength and fracture toughness of the scaffold, which, together with the strong hydrophilicity of the hydrogel, resulted in an alternative material for cartilage repair with similar properties to those of native cartilage [300].

In addition to achieving the function of ECM, another key issue in scaffold design is the ability to control the release of biological signals (mainly growth factors) that regulate cellular responses. Growth factors, such as BMPs, VEGF, and transforming growth factor-β (TGF-β), play a significant role in regulating chondrocyte metabolism and ECM formation [301]. However, due to its short half-life, the growth factors diminish rapidly upon entry into the body, thus often requiring repeated administration at concentrations well above physiological levels, which can easily lead to systemic impacts such as kidney and liver fibrosis, joint inflammation, synovial hyperplasia and osteophyte formation. As a result, achieving sustained controlled release of growth factors at the defective site by mimicking the in vivo injury repair process is a popular research topic for biomimetic cartilage tissue scaffolds. A simpler approach is to encapsulate growth factors in a certain carrier, such as microspheres [302, 303]. Much more sophisticated carriers are scaffolds that can reconstitute some of the ECM properties, including both natural and synthetic polymers, whose release rates are controlled either by diffusion of growth factors or by the degradation rate of material. The introduction of nanocomposite is vital in order to fabricate the optimal scaffold. For example, Lim et al. prepared a dual growth factor-loaded chitosan nanoparticle/alginate hydrogel system in which TGF-β_2_ was loaded on chitosan and BMP-7 on alginate. This system showed a rapid release of BMP-7 and a slow release of TGF-β_2_, providing growth factor delivery kinetics to promote chondrogenesis of MSCs and cartilage repair [304]. In another study, Shen et al. developed a GO bound scaffold of photopolymerizable poly-D, l-lactic acid/polyethylene glycol (PDLLA) hydrogel for the prolonged release of the chondroinductive growth factor TGF-β3. The results indicated that the incorporation of GO inhibited the burst release of TGF-β3 and significantly increased the releasing time of TGF-β3 in the hydrogel scaffold. Furthermore, after subcutaneous implantation in vivo, hBMSC-seeded TGF-β3-GO/PDLLA hydrogel constructs showed higher rates of chondrogenesis than those without GO (Fig. 7D) [305]. In addition, there are biomimetic scaffolds more similar to natural ECMs that release active substances based on the physicochemical state of the repair area, such as hydrogels that can reflect matrix pressure stimulation potentially providing mechanically controlled delivery of growth factors [306], which is more comparable to natural release process in vivo than the common concept of rapid release as well as release in a molecular storage manner.

### Engineered EVs

As previously mentioned, nano-scaffolds and -hydrogels have achieved some remarkable results as temporary extracellular matrices for therapeutic purposes, but the direct implantation of seed cells (MSCs) still involves challenges such as immune rejection [307] and potential tumorigenicity [308]. With the advancement of research, numerous studies indicate that apart from their differentiation capacity, stem cells also secrete paracrine factors that can reduce onsite cell apoptosis, improve cell survival, promote proliferation and differentiation [309, 310]. In particular, EVs (especially exosomes) have demonstrated their potential in tissue repair. In recent years, the role of different EV types in orthopedic diseases has been investigated intensively, and EV-based cell-free therapy is becoming a popular research topic.

**Fig. 8:**
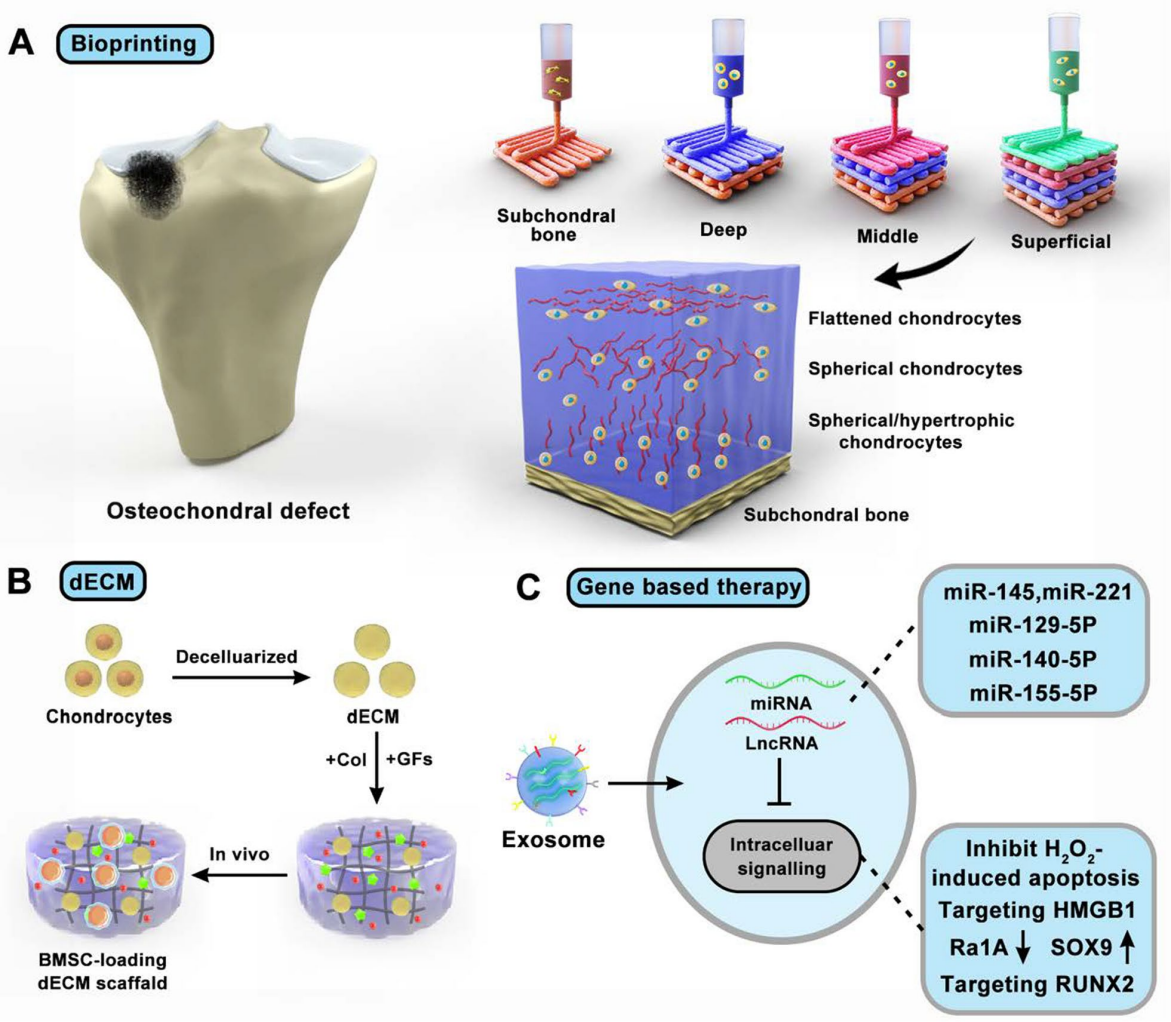
Promising nanotechnologies in cartilage repair. **A** Bioprinting enables multi-layer printing of cartilage tissue engineering scaffolds to reproduce their complex hierarchical structure. **B** ECM materials obtained by decellularization can self-assemble into gels that provide growth factors and seed cells for cartilage regeneration and repair. **C** Therapeutic cell-secreted exosomes can overexpress target mRNAs through genetic modification to directly activate or inhibit certain signalling pathways in target cells, alleviating the progress of OA

As intercellular and extracellular communicators, exosomes can directly activate or deactivate certain signalling in the target cells through binding to relevant receptors (Fig. 8C). They can also introduce genetic material into cells via endocytosis to promote certain proteins expression in the mediating cellular behaviors [311]. Specifically, therapeutic cells, especially MSCs, secrete exosomes that regulate the damaged tissue environment and coordinate the subsequent regenerative processes by transferring their bioactive substances [312]. Currently, the major studies focus on the loading components and mechanisms of exosomes derived from different tissue origin. For example, ADSCs-derived exosomes upregulated miR-145 and miR-221within periosteal cells and inhibited H_2_O_2_-induced apoptosis, resulting in enhanced proliferation and chondrogenesis [313]. Similarly, exosomal miR-136-5p secreted by BMSCs promoted chondrocyte migration in vitro, and prevented cartilage degeneration in vivo [314]. In another example, exosomal miR-129-5p from human synovial membrane-derived mesenchymal stem cells (SMSCs) inhibited the inflammatory responses and apoptosis by targeting the 3'-UTR of high mobility group protein-1 (HMGB1) to regulate IL-1β, which slow the progression of OA [315]. In addition, genetic modification can be used to modify the exosomal secretion and contents to obtain more desirable engineered exosomes and improve their therapeutic effect. For instance, to minimize the suppression of SOX9 expression induced by SMSCs-exosomes [316], miR-140-5p overexpressed SMSCs were fabricated using lentivirus transfection. SMSCs-miR140 exosomes no longer inhibited the SOX9 expression due to the downregulation of Ra1A, resulting in the upregulated cartilage-specific ECM deposition. More impressively, SMSCs-miR140 exosomes slowed the progression of OA as compared to SMSCs exosomes in an anterior cruciate ligament transection (ACLT) rat model. Similar result was concluded from Wang and his/her colleagues’ study [317], where exosomes from SMSCs with over expressed miR-155-5P enhanced the chondrogenesis of OA chondrocytes via targeting runt-related transcription factor 2 (RUNX2).

In addition to being used directly as biological agents for cartilage repair, or as bioactive coatings to functionalize the material surface for improved cell-implant interaction [198, 199], exosomes can also be embedded in biomimetic scaffolds for their controlled release in cartilage tissue engineering. For instance, Hu et al. demonstrated that human umbilical cord mesenchymal stem cells derived exosomes (hUC-MSCs-exosomes) containing GelMA/nanoclay hydrogel was effective in promoting cartilage regeneration. Further studies showed that hUC-MSCs-exosomes inhibited phosphatase and tensin homolog deleted on chromosome 10 (PTEN) levels. The further introduction of miR-23a-3p upregulated the expression of protein kinase B (AKT), resulting in the enhanced migration, proliferation and differentiation of BMSCs and chondrocytes [318]. In another study, Chen et al. designed an ECM/GelMA scaffold for MSC-exosome delivery and evaluated its ability to repair osteochondral defects using a rabbit model. The results showed that this biological scaffold effectively restored chondrocyte mitochondrial dysfunction. Meanwhile, the scaffold also promoted cell migration and contributed to the polarization of synovial macrophage to M2 phenotype, significantly improving cartilage regeneration [319].

## Future perspectives and conclusion

The development of nanomedicine has significantly changed the orthopedic surgery landscape. As outlined in this review, nanomedicine has been applied in different areas preclinically and clinically, including diagnosis, imaging, pharmaceutics and regenerative medicine (Table 3). The various delivery vehicles have also been developed to improve the therapeutic effect of nanomedicine, maximising the clinical translation potential. More importantly, through incorporating with existing surgical treatment and technologies, such as metal implants, nanomedicine could have a direct impact on bone and cartilage repair. That being said, limitations and challenges still exist, and issues need to be addressed in order to achieve satisfactory clinical outcomes.

**Table 3:** Specific nanotechnology applied in different disease models

Nanotechnology		Disease Model	Application
Nano-diagnostics	QD	Orthopedic implant-associated infection	Assessing the status of the implants
	Nanosensor	OA	Assessing the development of OA in a non-invasive and real-time manner
	AuNPs-based Biochip	Osteoporosis	Providing accurate identification of bone damage levels
	AuNPs-based MRI contrast agents	Fatigue-induced bone microdamage	Detecting bone mass and bone quality
	AuNP-based probe	OA	Detect ADAMTS-4 activity in the synovial fluid
Targeted drug delivery	AgNPs	Periprosthetic infections	Inhibiting bacteria
	MNP	Degenerative joint diseases	Sustained release of diclofenac sodium
	BCP NPs	Cranial critical-sized bone defects	Sustained release of BMP-2
	Liposomes	OA	Targeting the inflammation site with increased release of p-coumaric acid
	Polymeric micelles	Metastatic bone tumours	Targeted delivery of docetaxel and doxorubicin
	PNPs	RA	Targeted delivery of FK506
	BMSC-exosome	Osteoporosis	Increasing the osteogenic differentiation of BMSCs
	STExos	Femur fracture	Promoting the osteogenic differentiation of BMSC to accelerate bone healing
	Exosome	RA	Targeted delivery of Dex
Tissue engineering	Nanofibrous scaffolds	Bone/cartilage defects	Potential graft materials for orthopedic applications with outstanding advantages, including favourable biocompatibility, biodegradability, certain porosity and suitable strength
	Nanocomposite hydrogels	Bone/cartilage defects	Minimally invasive injectable with good dispersion of bioactive factors for matching irregular defects
	Surface modification	Fracture	Good choice as joint-replacement material due to low wear and high strength
	Nano-coating	Orthopedic implant-associated infection	Excellent material for long-term implant devices
	nHA	Fracture	Promoting tissue integration
	Ostim	THA	Reduce the penetration of bone cement
	Ta-coated electrospun PLA NFs	Fracture	Being fabricated into rods, screws and plates
	Engineered EVs	OA	Mediating intercellular communication and regulating the damaged tissue environment

Although nanocomposite hydrogels have made a significant impact in tissue engineering, they are often deficient in macroscopic pores and complex microvascular systems, which impacts the transport of nutrients and discharge of metabolites, leading to a considerable loss in the viability and function of seeded cells. 3D porous nanostructured scaffolds with high porosity and suitable pore size overcome the limitation of hydrogels, but most of their internal pores are closed pore structures with limited pore connectivity, resulting in reduced cell infiltration, migration and tissue growth. The development of 3D printing and biofabrication has brought landmark advances in tissue engineering for bone and cartilage repair compared to traditional process methods [320]. Based on its advantages of being able to print layer-by-layer and precisely regulated, the bio-3D printed bone/cartilage tissue-engineered scaffolds have achieved the advancement from single-layer [321] to multi-layer [322] and the leap from pure cartilage to osteochondral integration [323], which both mimic the multi-phase material of native bone/cartilage and reproduce their complex hierarchical structure (Fig. 8A). More importantly, the 3D printing technology allows precise control over the scaffold structure to match with patient anatomical structure, ensuring the personalised treatment to achieve maximum therapeutic potential. However, the adoption of 3D bioprinting into cartilage and bone engineering could be particularly challenging, due to the high mechanical requirement for the fabricated scaffolds. Although thermoplastic scaffolds offer adequate mechanical properties, they often cannot encapsulate cells with limited cell intrusive capacity to promote cellular function, resulting in insufficient tissue regeneration [153]. Bioink (hydrogel) based scaffold, on the other hand, can provide a benign microenvironment for cellular proliferation and differentiation but offer inadequate mechanical strength [280]. That being said, the recently developed hybrid biofabrication approach allows the fabrication of bone draft with enhanced mechanical properties (PCL scaffold with magnesium composite) and improved osteogenesis (hydrogel-based bioink and strontium nanocomposite), which unravels the ultimate potential of 3D printing in bone engineering [324]. In addition, active factors such as TGF and BMP are gradually being added to 3D printing inks for bio-stimulation by researchers to induce region-specific differentiation and growth of cells in different stratifications of the scaffold for optimal repair [325]. However, the ECM of native bone and cartilage is a complex microenvironment with chemical stimuli (such as growth factors) and mechanical stimuli [326]. Therefore, it is necessary to construct an environment that mimics the in vivo stimulation to impart biological activity to the scaffold structure and to promote its functionalization.

Decellularized extracellular matrix (dECM) is a new direction in developing biomimetic biomaterials. Decellularization refers to the effective removal of DNA, RNA and other components from tissues or organs while protecting the composition and structural integrity of the natural ECM, thus achieving the goal of avoiding rejection reactions caused by cellular antigens in xenografts and allografts [327, 328]. The ECM materials obtained by decellularization not only have reduced immunogenicity, but also retain a large amount of natural ECM active components (e.g. collagen, GAGs, growth factors), which can be self-assembled into gels to act as injectable scaffolds for tissue regeneration and repair, thereby delivering growth factors and seed cells [329–331] (Fig. 8B). It has been demonstrated that the reconstruction of corresponding tissues using homologous dECM scaffolds can promote tissue regeneration and repair more effectively than those from other tissue sources [332]. Therefore, dECM from bone/chondral tissue is undoubtedly the most suitable natural material for bone/chondral tissue regeneration. Pati et al. used a combination of cartilage dECM and PCL to construct cartilage structures with stable and high mechanical strength [333]. Similarly, Beck et al. created methacrylated solubilized decellularized cartilage gels with an elastic compressive modulus close to that of native articular cartilage (1070 ± 150 kPa) [334]. In addition to structural and compositional similarities to the natural ECM, dECM induces proliferation and differentiation of stem cells in the host. Luo et al. inoculated human infrapatellar fat pad derived stem cells on a cartilage dECM scaffold and observed enhanced cell proliferation [335]. In another study, Choidn et al. examined the efficacy of an electrospun PCL based fibrous scaffold embedded with bone dECM. The results showed that dECM had little effect on the mechanical properties of the scaffold compared to PCL, but significantly affected cell adhesion and proliferation as well as differentiation [336]. On the other hand, Lu et al. compared the differences between two collagen scaffold systems (in the forms of particle and solution, respectively) functionalized with porcine dECM. Although both forms of dECM supported MSCs recruitment, proliferation and promotion of chondrogenic differentiation, better performance was seen in the latter group, reflecting a clear difference in the local cellular microenvironment provided by the dynamic regulation of biological components in the two forms of scaffolds over time, which suggested that the optimization of suitable processing methods should be taken into account when designing dECM-based scaffolds [337]. To sum up, dECM shows the prototype of a novel bioactive natural scaffold material in bone and cartilage tissue engineering. However, some studies have reported that excessive decellularization treatment would weaken the biological function of ECM, while there is no significant difference in the biological activity of dECM obtained after thorough versus relatively incomplete decellularization treatment [338]. In addition, the existing decellularization methods have unavoidable adverse effects on ECM, which requires further improvement of dECM mechanical, structural and biological properties during the subsequent preparation to compensate for the ECM damage caused by decellularization [334, 339]. Moreover, it is a challenge to improve the reproducibility of dECM batches while scaling up production.

Gene-based therapy would be the future for cartilage and bone repair as it targets the mechanisms associated with a specific disease, treating the causes rather than the symptoms. In order to achieve this goal, a safe and efficient delivery system is necessary. Despite the high transfection efficiency of viral vectors used by most gene-based therapies, its limitations, such as immunogenicity and cytotoxicity [340], significantly impact the success of clinical translation. Recent advances in nanomedicine have given rise to a range of non-viral gene carriers that are currently in different stages of preclinical trials. For example, the peptide-NF-κB siRNA nanoparticles developed by Yan et al. were demonstrated to penetrate into chondrocytes freely and persist for more than 2 weeks, which maintained cartilage homeostasis by enhancing AMPK signalling while inhibiting mTORC1 and Wnt/β-catenin activity, thereby controlling post-injury cartilage responses (such as chondrocyte apoptosis, reactive synovitis) [341]. PLGA-based nanoparticles have also been used for the gene therapy of cartilage injury. Shi et al. successfully transfected BMP-4 plasmids into rabbit ADSCs using PLGA nanoparticles, and showed through further experiments that scaffolds seeded with this nanocomplex could effectively promote chondrogenesis in vitro and in vivo [342]. In addition, a relatively new class of siRNA delivery systems, namely liposomal NPs, is expected to be a powerful tool for treating cartilage damage by knocking down specific genes because of their ability to effectively transfect 100% of chondrocytes [343].

Although nanomaterial-based therapeutic products have been emerging in orthopedic fields such as drug delivery, biosensors and tissue-engineered scaffolds, the sample size of related studies is currently insufficient with a lack of long-term follow-up. Given that previous studies have focused on blending different materials to optimize the quality of implant-host integration, issues related to nanotoxicity and inflammatory responses cannot be ignored and require more preclinical studies for safety verification. In addition to continuously improving the match between nano-design and manufacturing processes (e.g., increasing the development of nano-materials compatible with 3D printers, and introducing the computer-aided design together with finite element analysis further to understand the relationship between scaffold structure and mechanical properties), it is more important to strengthen the cooperation between clinicians and laboratory staff, in order to provide solutions for the key problems in the practical application of tissue-engineered structures, accelerating the advancement of nanomedicine from the bench to the operating room or even the bedside.

## Data Availability

Data sharing is not applicable to this article as no datasets were generated or analysed during the current study.

## Abbreviations

TJR: Total joint replacement
ECM: Extracellular matrix;
NPs: Nanoparticles
EVs: Extracellular vesicles
POCT: Point⁃of⁃care testing
BCA: Bio-barcode assay
QD: Quantum dot
LFIA: Lateral flow immunoassay
IL-6: Interleukin-6
ELISA: Enzyme-linked immunosorbent assay
NO: Nitric oxide
OA: Osteoarthritis
AFM: Atomic force microscopy
BSU: Bone structural units
PET: Positron emission computed tomography
MRI: Magnetic resonance imaging
RES: Reticuloendothelial system
PEG: Polyethylene glycol
NDDSs: Nano drug delivery systems
AuNPs: Gold nanoparticles
SPR: Surface plasmon resonance
ADAMTS-4: A disintegrin and metalloproteinase with thrombospondin motif-4
ACAN: Aggrecan
AgNPs: Silver nanoparticles
ROS: Reactive oxide species
MNPs: Magnetic nanoparticles
MDT: Magnetic drug targeting
NSAID: Non-steroidal anti-inflammatory drug
BMSCs: Bone marrow mesenchymal stem cells
sGAG: Sulfated glycosaminoglycan
SPIOs: Super paramagnetic iron oxides
CNTs: Carbon nanotubes
NDs: Nanodiamonds
CDs: Carbon dots
MWCNTs: Multi-walled carbon nanotubes
ALP: Alkaline phosphate
AMSCs: Adipose-derived mesenchymal stem cells
MSNs: Mesoporous silica nanoparticles
ALN: Alendronate
HA: Hydroxyapatite
CaP: Calcium phosphate
BMP-2: Bone morphogenetic protein-2
BCP: Biphasic calcium phosphate
VEGF: Vascular endothelial growth factor
ML: Mannose incorporated liposomal delivery system
CA: P-coumaric acid
AIA: Adjuvant-induced arthritic
NFATc1: Nuclear factor of activated T-cells c1
PAMAM: PEGylated polyamidoamine
IGF-1: Insulin-like growth factor 1
DOX: Doxorubicin
DEX: Dextran
DSPE: 1,2-Distearoyl-sn-glycero-3-phosphoethanolamine
PDPA: Poly (2-diisopropylaminoethyl methacrylate)
ABC: Accelerated blood clearance
CARPA: Complement activation-related pseudo allergy
RBCs: Red blood cells
CIA: Collagen-induced arthritis
PNPs: Platelet-mimetic nanoparticles
RA: Rheumatoid arthritis
MVs: Microvesicles
PTX: Paclitaxel
Ti: Titanium
siRNA: Small interfering ribonucleic acids
HEK-293: Human embryonic kidney cells
PANC-1: Human pancreatic adenocarcinoma
ST: Stromal cell
OVX: Ovariectomy
FA: Folic aci
Chol: Cholesterol
OBs: Osteoblasts
OCs: Osteoclasts
EC: Endothelial cell
COL-I: Collagen I
3D: Three-dimensional
NFs: Nanofibers
CS: Chitosan
PLA: Poly (lactic acid)
PGA: Poly (glutamic acid)
PCL: Poly (ε-caprolactone)
RGD: Arginine-glycine-aspartate
EO: Endochondral ossification
MNF: Macroporous nanofibrous scaffold
PLLA: Poly (L-lactic acid)
EYA: Sequential yarns manufacture and honeycombing
nβ-TCP: Nano-sized β-tricalcium phosphate
PLGA: Poly- (lactide-co-glycolic)
nHA: Nano-hydroxyapatite
SBF: Simulated body fluid
TIPS: Thermally induced phase separation
GelMA: Methacrylated gelatin
MAPK: P38 mitogen-activated protein kinase
ERK: Extracellular signal-related kinase
RNT: Rosette nanotube
pHEMA: Poly (2-hydroxyethyl methacrylate)
PIC: Polyion complex
SD: Sprague–Dawley
PECE: PEG-PCL-PEG copolymer
MBGNs: Mesoporous bioactive glass nanoparticles
NAGA: (N-acryloyl glycinamide)
CAM: Chicken chorioallantoic membrane
CHPOA: Cholesteryl group- and acryloyl group-bearing pullulan
FGF18: Fibroblast growth factor 18
NiPAAM: N-isopropylacrylamide
LCST: Lowest critical solution temperature
CoCrMo: Cobalt-chromium–molybdenum
TiO_2_: Titanium oxide
PEO: Plasma electrolytic oxidation
LPEB: Large pulse electron beam
SPD: Severe plastic deformation
P/M: Powder metallurgy
Li: Lithium
MAO: Micro-arc oxidation
DEVs: Secreted by decidual mesenchymal stem cells
Mg: Magnesium
TPA: Transonic particle acceleration
ECAD: Electrochemical assisted deposition
BG: Bioactive glass
Ta: Tantalum
DC: Direct current
PMMA: Poly (methyl methacrylate)
CPC: Calcium phosphate cement
CDHA: Calcium-deficient hydroxyapatite
PP: Polypropylene
rGO: Reduced graphene oxide
GMA: Glycidyl methacrylate
MWT: Microwave technique
CVD: Chemical vapour deposition
PVD: Physical vapour deposition
EPD: Electrophoretic deposition
FGF-2: Fibroblast growth factor-2
ACI: Autologous chondrocyte transplantation
MACI: Matrix-induced autologous chondrocyte implantation
PVA: Poly (vinyl alcohol)
BSA: Bovine serum albumin
CMs: Chitosan-based microspheres
CMC: Carboxymethyl chitosan
OCS: Oxidized chondroitin sulfate
PLCL: Poly (lactide-co-E-caprolactone)
SF: Silk fibroin
DCECM: Decellularized cartilage extracellular matrix
TGF-β: Transforming growth factor-β
PDLLA: Poly-D, l-lactic acid/polyethylene glycol
HMGB1: High mobility group protein-1
ACLT: Anterior cruciate ligament transection
RUNX2: Runt-related transcription factor 2
PTEN: Phosphatase and tensin homolog deleted on chromosome 10
AKT: Protein kinase B
